# New Pyrrole Derivatives as Promising Biological Agents: Design, Synthesis, Characterization, In Silico, and Cytotoxicity Evaluation

**DOI:** 10.3390/ijms23168854

**Published:** 2022-08-09

**Authors:** Beatrice-Cristina Ivan, Stefania-Felicia Barbuceanu, Camelia Mia Hotnog, Adriana Iuliana Anghel, Robert Viorel Ancuceanu, Mirela Antonela Mihaila, Lorelei Irina Brasoveanu, Sergiu Shova, Constantin Draghici, Octavian Tudorel Olaru, George Mihai Nitulescu, Mihaela Dinu, Florea Dumitrascu

**Affiliations:** 1 Faculty of Pharmacy, “Carol Davila” University of Medicine and Pharmacy, 6 Traian Vuia Street, 020956 Bucharest, Romania; 2 Center of Immunology, “Stefan S. Nicolau” Institute of Virology, Romanian Academy, 285 Mihai Bravu Ave., 030304 Bucharest, Romania; 3 Laboratory of Inorganic Polymers, “Petru Poni” Institute of Macromolecular Chemistry, Aleea Grigore Ghica Voda, 41A, 700487 Iasi, Romania; 4 “C.D. Nenitescu” Institute of Organic and Supramolecular Chemistry Romanian Academy, 202B Splaiul Independenței, 060023 Bucharest, Romania

**Keywords:** pyrroles, dipolarophile alkynes, [3 + 2] cycloaddition, X-ray diffraction, cytotoxicity, antitumor activity

## Abstract

The current study describes the synthesis, physicochemical characterization and cytotoxicity evaluation of a new series of pyrrole derivatives in order to identify new bioactive molecules. The new pyrroles were obtained by reaction of benzimidazolium bromide derivatives with asymmetrical acetylenes in 1,2-epoxybutane under reflux through the Huisgen [3 + 2] cycloaddition of several ylide intermediates to the corresponding dipolarophiles. The intermediates salts were obtained from corresponding benzimidazole with bromoacetonitrile. The structures of the newly synthesized compounds were confirmed by elemental analysis, spectral techniques (i.e., IR, ^1^H-NMR and ^13^C-NMR) and single-crystal X-ray analysis. The cytotoxicity of the synthesized compounds was evaluated on plant cells (i.e., *Triticum aestivum* L.) and animal cells using aquatic crustaceans (i.e., *Artemia franciscana* Kellogg and *Daphnia magna* Straus). The potential antitumor activity of several of the pyrrole derivatives was studied by performing in vitro cytotoxicity assays on human adenocarcinoma-derived cell lines (i.e., LoVo (colon), MCF-7 (breast), and SK-OV-3 (ovary)) and normal human umbilical vein endothelial cells (HUVECs). The obtained results of the cytotoxicity assessment indicated that the tested compounds had nontoxic activity on *Triticum aestivum* L., while on *Artemia franciscana* Kellogg nauplii, only compounds **2c** and **4c** had moderate toxicity. On *Daphnia magna*, **4b** and **4c** showed high toxicity; **2a**, **2b**, and **2c** moderate to high toxicity; only **4a** and **4d** were nontoxic. The compound-mediated cytotoxicity assays showed that several pyrrole compounds demonstrated dose- and time-dependent cytotoxic activity against all tested tumor cell lines, the highest antitumor properties being achieved by **4a** and its homologue **4d**, especially against LoVo colon cells.

## 1. Introduction

Cancer is one of the most feared diseases, being the leading cause of death worldwide. Colorectal, breast, and ovarian malignancies are among the most common human cancers in the world and also the most diagnosed cancers in Europe and the United States, with high incidence and mortality [1]. Although various drugs are currently known to treat this disease, there is still the risk of some tumors developing resistance to the chemotherapeutic drugs used, thus leading to ineffective therapy [2]. For this reason, it is imperative to discover new molecules with antitumor action that are much more targeted and efficient, with increased bioavailability and smaller side effects than those currently used.

Heterocycles, cyclic compounds having one or more heteroatoms, represent one of the most important classes of organic compounds for medicinal chemistry, many of them being essential to life because of their vital role in the metabolism of living cells [3]. Within the heterocyclic compounds class, nitrogen heterocycles occupy a special place, being the most common structural skeleton of drugs on the market [4,5]. In particular, pyrroles, five-membered heterocycles with one nitrogen atom, are a class of natural or synthetic compounds that receive special attention from researchers because they are an important source of new bioactive compounds, thus occupying a significant position in drug development programs [4]. Due to the fact of its aromatic structure, pyrrole can react with various electrophiles, leading to a wide variety of compounds with biological potential, some of them having a crucial role in life processes. Numerous pyrrole derivatives have been reported to have various biological activities [6,7,8,9,10,11,12,13,14], especially anticancer properties [15,16,17,18,19,20,21,22].

**Figure 1:**
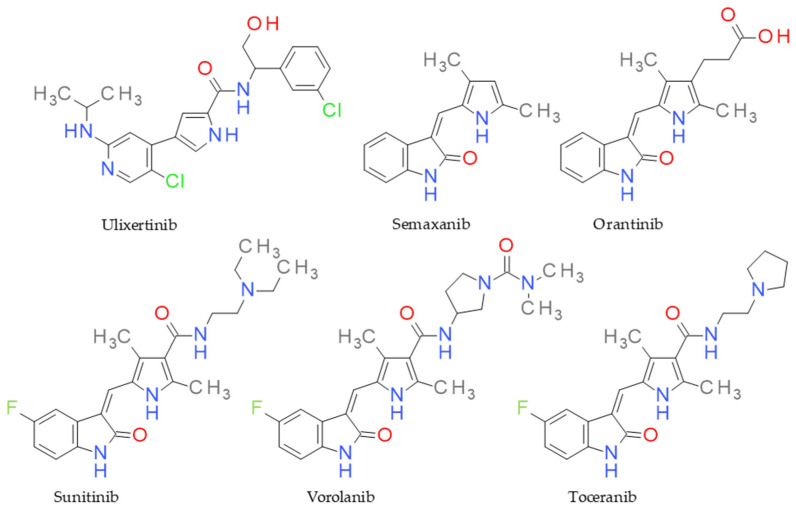
Structure of anticancer drugs sharing the pyrrole ring.

Functionalized pyrrole scaffolds are important chemotypes for designing protein kinase inhibitors and have excellent antiproliferative potential [23]. For example, sunitinib is a multitargeted receptor tyrosine kinase inhibitor, being used as a first-line therapy in advanced renal cell carcinoma [24,25]. Toceranib is a derivative of sunitinib with antiangiogenic and antiproliferative effects via inhibiting several receptor tyrosine kinases and is approved for veterinary use [26]. Vorolanib, known as X-82, is an oral protein kinase inhibitor targeting the vascular endothelial growth factor receptor (VEGFR), platelet-derived growth factor receptor (PDGFR), and colony-stimulating factor receptor (CSF) [27]. Ulixertinib is another example of a pyrrole-based protein kinase inhibitor with high potency and selectivity towards ERK1/2 (extracellular signal-regulated protein kinase), and it is approved as a treatment for cancer that depends on the MAPK pathway [28]. Semaxanib is also a protein kinase inhibitor that targets the VEGF pathway, producing antiangiogenic effects [29] (Figure 1). In addition, other antitumoral agents containing a pyrrole core are used in therapy or are in clinical studies [22].

Regarding the synthesis of pyrroles, there are various synthetic procedures that can be used including the classical Paal–Knorr and Hantzsch reactions [30,31,32]. An interesting method of obtaining pyrroles is via the reaction of benzimidazolium salts with dipolarophile alkyne derivatives [33,34]. Studies on the structure–biological activity relationship indicate that the carbonyl group in pyrrole molecules is a key moiety with an important role in biological activity [35]. Motivated by these aforementioned findings and in continuation of our previous research [34,35,36], we synthesized a series of new trisubstituted pyrrole derivatives with a carbonyl group by the reaction of benzimidazolium bromide with acetylenic dipolarophiles. The compounds were designed to target VEGFR and PDGFR, based on their structural similarities with sunitinib, vorolanib, and orantinib. The cytotoxic effect of the synthesized compounds was evaluated on the *Triticum aestivum* L. wheat species and the *Artemia franciscana* Kellogg and *Daphnia magna* Straus crustaceans. Moreover, an in vitro investigation of the potential antiproliferative activity of several pyrrole derivatives against solid tumor-derived cells was conducted using the human colon LoVo, breast MCF-7, and ovary SK-OV-3 cell lines, and their cytotoxic activity was compared to that induced by cisplatin (Cis-Pt), 5-fluorouracyl (5-FU), and doxorubicin (Dox).

## 2. Results and Discussion

### 2.1. Chemistry

The pyrroles obtained by reacting benzimidazolium or quinazolinonium salts with various dipolarophile agents (electron-deficient alkynes or alkenes derivatives) in the presence of base is a method that has attracted the interest of researchers in the heterocycle chemistry field. Depending on the nature of the salt heterocycle (i.e., benzimidazole or quinazolinone), the radical from the third position on the imidazole nucleus or the first position on the pyrimidine nucleus, and the reaction conditions, pyrroles or fused-pyrrole derivatives (i.e., pyrrolobenzimidazoles or pyrroloquinoxaline) can be obtained [32,34,37,38,39]. In the case of benzimidazole derivatives, if a fragment containing a methylenester or ketomethylene group is grafted in the third position, by varying the reaction conditions and the basic medium, condensed pyrrole derivatives are obtained [40]. Only in the case of some benzimidazole salts, was the pyrrole derivative isolated [33,34]. Other studies have shown that if the 1,3-dipolar cycloaddition reaction takes place using a benzimidazole salt with a cyanomethylene group attached to the nitrogen atom at the third position, pyrrole derivatives can be obtained as the majority [32] or as a unique product [34,36].

The new pyrroles were synthesized by the 1,3-dipolar cycloaddition reaction of some benzimidazole salts containing a cyanomethylene moiety with various dipolarophile alkynes, under reflux, in 1,2-epoxybutane acting as both the reaction medium and reagent for the generation of the benzimidazolium N-ylides intermediates.

**Scheme 1:**
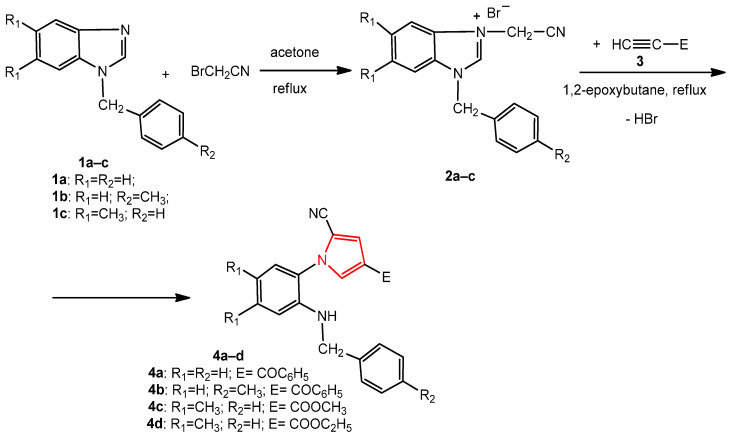
The synthesis of the new pyrroles, **4a**–**d**, from benzimidazole derivatives.

The synthesis of the new 1-benzyl-5,6-dimethyl-3-cyanomethylbenzimidazolium bromide **2c** was carried out through the alkylation of 1-benzyl-5,6-dimethylbenzimidazole **1c** with bromoacetonitrile, under reflux, in acetone (Scheme 1). The 1-(benzyl/4-methylbenzyl)-3-cyanomethylbenzimidazolium bromides **2a**,**b** have been synthesized using the same procedure previously [32,34]. The *N*-substituted benzimidazoles **1a**–**c** were obtained by the alkylation of benzimidazole and 5,6-dimethylbenzimidazole with benzyl chloride or 4-methylbenzyl chloride [34].

**Scheme 2:**
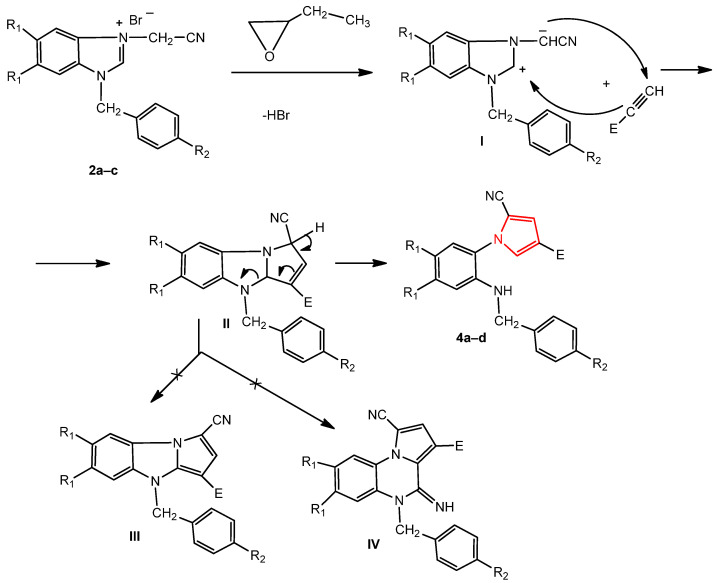
The probable mechanism for the formation of the new pyrroles **4a**–**d**.

The reaction mechanism of benzimidazole bromide and the acetylene derivative consisted, in the first stage, of an attack by the bromide ion from the corresponding salt, **2a**–**c**, on the epoxybutane with the opening of its ring, obtaining an alkoxide ion. Subsequently, the alkoxide ion attacked the methylene carbon of the cyanomethylene group, generating the corresponding ylide **I**. In the next stage, the ylide reacted with the alkyne derivative by a [3 + 2] dipolar cycloaddition reaction, obtaining the dihydropyrolobenzimidazole primary adduct **II**, leading to the pyrrole **4a**–**d** (by opening the imidazole ring) and not to the pyrrolobenzimidazole **III** or 4-iminopyrrolo [1,5-a]quinoxaline **IV** derivatives (Scheme 2) [32,34,38].

The structure of the new compounds was established via recorded spectral data (i.e., IR, ^1^H-NMR, and ^13^C-NMR), single-crystal X-ray diffraction analysis in the case of pyrrole **4d**, and by elemental analyses. The IR spectrum of the salt **2c** confirmed its structure by the presence of a new band at 2254 cm^−1^ characteristic of the cyano group. The ^1^H-NMR spectrum of this compound confirmed the reaction of 5,6-dimethylbenzimidazole with bromoacetonitrile by the presence of a new singlet signal due the presence of methylene protons linked to the cyano group at 5.97 ppm. In the ^13^C-NMR spectrum, the two representative signals at 35.0 ppm and 114.2 ppm, due to the same methylene and cyano carbons, are more proof of the obtainment of this new salt. The IR spectra confirmed the structure of the new pyrroles **4a**–**d** by the presence of a new absorption band from the 3358 to 3427 cm^−1^ region, which is characteristic of amino group stretching vibration. In addition, another new absorption band, due to the carbonyl group, was found in the range of 1632–1708 cm^−1^. The stretching band of the nitrile group (ν_CN_) appeared in the region 2216–2301 cm^−1^. The ^1^H-NMR data for compounds **4a**–**d** provide good evidence for their pyrrole structure and regioselectivity of [3 + 2] cycloaddition reaction between benzimidazolium *N*-ylides **I** (Scheme 2) and terminal alkynes (HC≡C-E). The main characteristic features in the ^1^H-NMR spectra were signals of the protons H-3 and H-5 of the pyrrole ring, which appeared as two doublets in the region of 7.31–7.52 ppm, with a coupling constant of ca. 1.7 Hz. The NH group appeared as a broad singlet or triplet due to the fact of its coupling with benzylic protons. In the case of compound **4d**, H-3 appeared as a doublet at 7.33 ppm, whereas the doublet corresponding to H-5 appeared at 7.47 ppm. The methylene protons of the ethyl and benzyl groups (i.e., CH_2_Ph and CH_2_O) overlapped and were in the range 4.26–4.33 ppm. The two methyl groups attached to the 1,2,4,5-tetrasubstituted benzene ring appeared as two singlets at 2.16 and 2.21 ppm, whereas the protons of the methyl radical from the ethyl group presented as a triplet at 1.33 ppm, with a coupling constant of 7.1 Hz. The protons of the phenyl moiety were superimposed on a multiplet, with chemical shifts in the range 7.25–7.32 ppm. The chemical shifts of the singlets from 6.57 to 6.89 ppm were attributed to the protons from the tetrasubstituted benzene ring. The ^13^C-NMR spectra highlighted the pyrrole core through the carbon signals from 121.6. to 122.5 ppm (C-3), 107.3–125.8 ppm (C-4), and 131.7–132.8 (C-5). The new signals, characteristic of the C=O carbon atom, which were identified at δ 188.8–188.9 ppm in the benzoyl-pyrroles **4a** and **4b** and at 162.8–163.2 ppm in the ester pyrroles **4c** and **4d**, the most deshielded carbon, are good proof of the formation of these compounds. The other signals of the proton and carbon atoms from the new compounds were present in the NMR spectra at the expected values of the chemical displacement. Regarding the single-crystal X-ray diffraction study for **4d**, the crystallographic data and refinement parameters are provided in Table 1.

**Figure 2:**
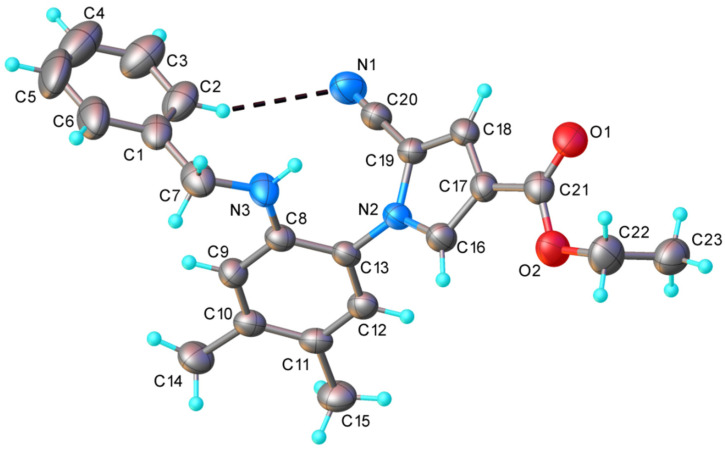
X-ray molecular structure of compound **4d** with atom labeling and thermal ellipsoids at the 40% level. Intramolecular H-bond parameters: C2-H···N1 [C2-H 0.93 Å, H···N1 2.73 Å, C2···N1 3.608 Å, ∠ C2HN1 158.3°].

The results of the single-crystal X-ray diffraction study on **4d** are depicted in Figure 2. This compound exhibited a molecular crystal structure comprising two crystallographically independent but chemically identical neutral entities (denoted as A and B).

**Figure 3:**
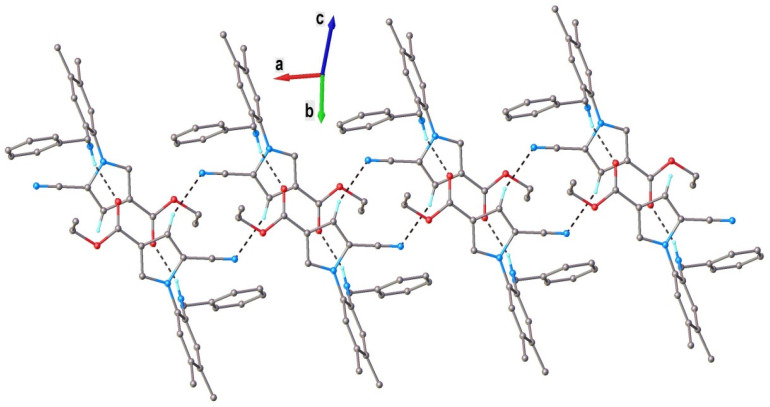
A view of a 1D supramolecular chain in the crystal structure of **4d** (for A molecules). Nonrelevant H atoms were omitted. H-bonds parameters: N3-H···O1 [N3-H 0.86 Å, H···O1 2.15 Å, N3···O1(1 − x, −y, 1 − z) 2.873(3) Å, ∠ N3HO1 141.3°]; C18-H···N1 [C18-H 0.93 Å, H···N1 2.59 Å, C18···N1(−x, −y, 1 − z) 3.384(4) Å, ∠ N3HO1 144.0°].

Both independent molecules presented a nonplanar configuration, which was stabilized via weak intramolecular C2-H···N1 hydrogen bonding. The dihedral angles, formed by a C8A/C13A ring with C1/C6 and N2/C16/C19 cycles, were 85.0(1)° and 66.5(1)° (for molecule A) and 90.2(1)° and 64.0(1)° (for molecule B). The neutral molecules interacted in the crystal through N-H···O and C-H···O H-bonds, which determined the formation of the two crystallographically independent 1D supramolecular array. As an example, a view of a supramolecular chain comprising A molecules is shown in Figure 3.

### 2.2. Toxicity Evaluation

#### 2.2.1. Plant Toxicity Assay

**Figure 4:**
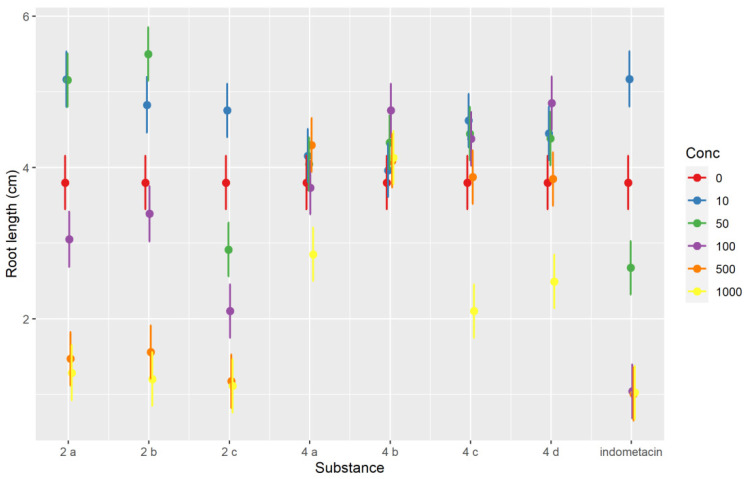
Interaction plot showing the interaction effects between the tested compounds and the concentration for the Triticum phytotoxicity test.

**Figure 5:**
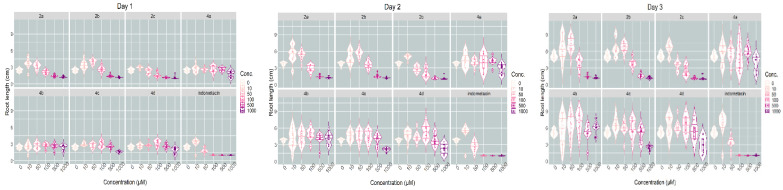
Violin plots showing the variations in Triticum’s main rootlet length under the influence of the substances tested at different concentration levels and day of measurement.

The robust mixed-effects model used explained approximately three-quarters of the variability observed (conditional R2 of 0.732) and indicated that the root length was dependent on the day of measurement (it increased with each day, *p* < 0.001), but there were many strong (*p* < 0.001) or moderate (*p* < 0.05) interactions between compounds and concentrations. The sense of the interactions between the two variables can be inferred from the interaction plot (Figure 4). Most compounds evaluated inhibited Triticum growth at the highest concentration level (1000 μM). The inhibition of root development can be an indicator of a compounds’ antiproliferative effect [41]. Whereas root length was strongly dependent on the concentrations used for indomethacin and the salts **2a**–**2c**, in the case of pyrroles **4a**–**4d**, the inhibitory effect was much less pronounced, particularly at concentrations lower than 500 μM, but even at 500 and 1000 μM the difference was sizeable. The indomethacin exerted the strongest inhibitory effect. As shown by the interaction plot, salts **2a**–**2c** tended to have slight stimulatory effects at low concentration levels (10–50 μM), whereas such an effect was not seen for **4a**–**4d** (Figure 4). Variations in Triticum’s main rootlet length under the influence of the compounds tested at different concentration levels and the day of measurement are represented in Figure 5.

**Figure 6:**
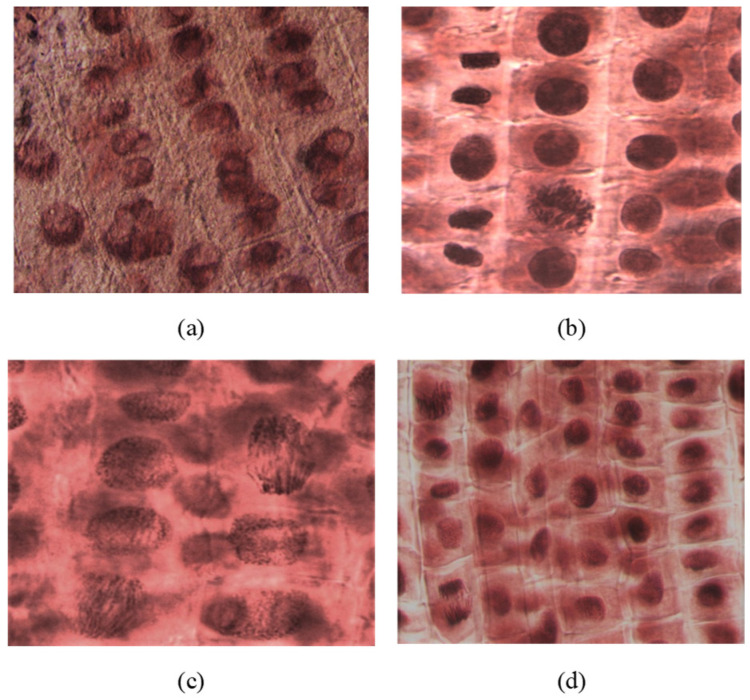
(**a**) Nuclei with hypertrophied nucleoli observed in rootlets treated with compound **2c** (concentration of 1000 μM); (**b**) metaphase in tropokinesis and normal telophases observed in rootlets treated with compound **4c** (concentration of 1000 μM); (**c**) telophase with chromosomal bridges observed in rootlets treated with compound **4a** (concentration of 10 μM); (**d**) telophase with delayed chromosomes, metaphases, and interphases observed in rootlets treated with compound **2b** (concentration of 100 μM). All microphotographs were taken with a lens magnified by 40×.

The microscopic analysis showed that the compounds and concentrations with an inhibitory effect on root growth also had an effect on inhibiting cell division. Thus, areas of mitoinhibition, a nucleus with hypertrophied nucleolus, were observed (**2c** at 1000 μM). At low concentrations (100–10 μM), the number of dividing cells observed was higher. Tropokinesis, chromosomal bridges, or delayed chromosomes were also observed in some cases (e.g., **4c**-I, **4a**-V, **2b**-III; Figure 6).

#### 2.2.2. Animal Toxicity Assay

##### *Artemia franciscana* Toxicity Assay

**Figure 7:**
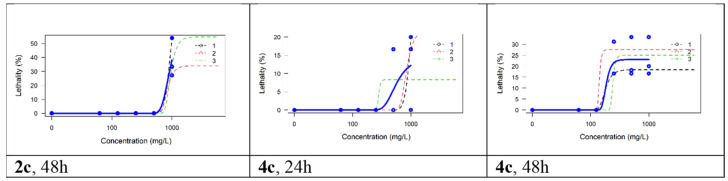
The lethality curves for the replicates and global models for compounds **2c** and **4c**.

All compounds evaluated manifested little toxicity on Artemia nauplii. Neither indomethacin nor **2a, 2b, 4a, 4b**, or **4d** caused any lethality in this test. The only lethality observed was for compounds **2c** and **4c**, but even in this case, it manifested only at the highest exposure (i.e., 1000 μM for **2c** and 250–100 μM for **4c**) and only after 48 h. Because for both compounds the lethality did not reach 50% at any concentration, the LC_50_ could only be estimated by extrapolation, which is methodologically problematic [42]; therefore, we only estimated the LC_25_: 793.32 μM for **2c** (equivalent to 282.6 mg/L) and 168.82 μM for **4c** (equivalent to 60.7 mg/L). The lethality curves for the replicates and global models for these two compounds are shown in Figure 7. In the toxicological literature on Artemia nauplii, concentrations have commonly been expressed in mg/L (ppm), and for known organic pollutants, such as bisphenol A, an LC_50_ of 34.45 mg/L was estimated at 48 h, whereas for sodium dodecyl sulfate (SDS), an LC_50_ of 7.87 mg/L was estimated [43]. The higher values observed in our experiment indicate that these two compounds (i.e., **2c** and **4c**) have lower acute toxicity than bisphenol A or SDS. For organo-phosphoric compounds, such as malathion, LC_50_ values of 1 to 17 mg/L were determined at 48 h, whereas for glyphosate LC_50_ point estimates were lower than 1 mg/L [44]. To conclude, most of the compounds synthesized and evaluated in this paper were devoid of toxicity on Artemia nauplii, and the only two that manifested some toxicity seemed to be less toxic than those used widely in industrial compounds such as bisphenol A or SDS.

##### *Daphnia magna* Toxicity Assay

**Figure 8:**
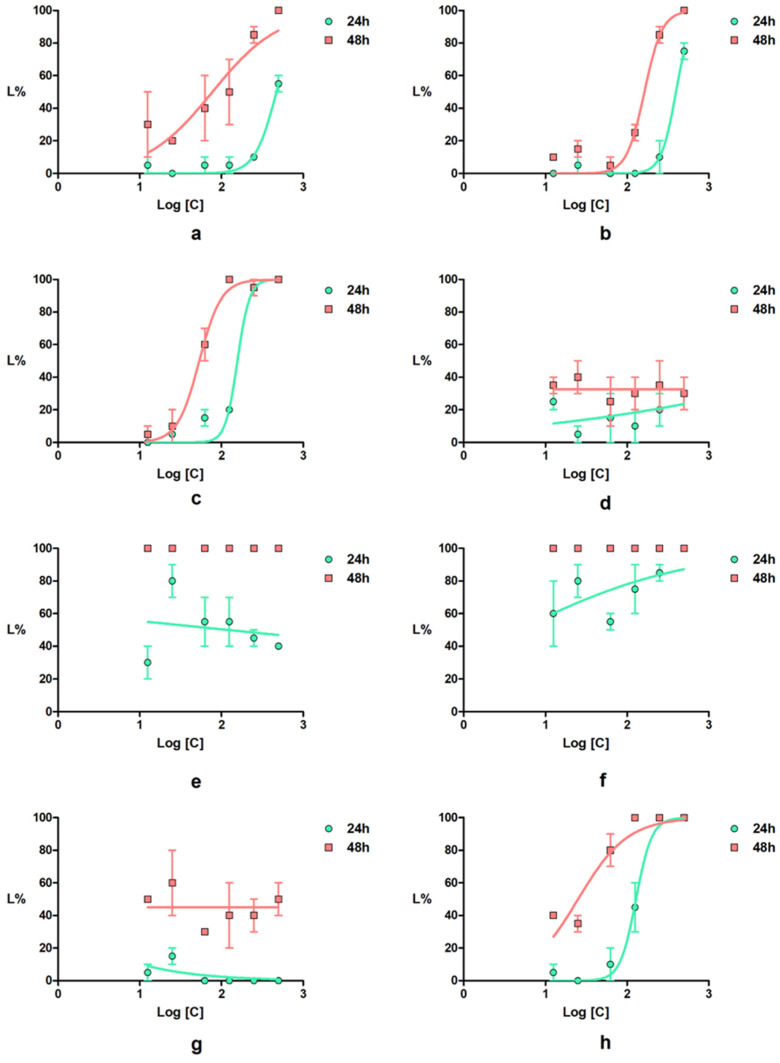
*Daphnia magna* lethality curves for the tested compounds: (**a**) **2a**; (**b**) **2b**; (**c**) **2c**; (**d**) **4a**; (**e**) **4b**; (**f**) **4c**; (**g**) **4d**; (**h**) indomethacin; error bars represent the standard error of the mean.

The results of *Daphnia magna* bioassay are presented in Table 2 and the lethality curves in Figure 8. The Daphnia magna toxicity induced by the salts **2a**–**2c** and by indomethacin were correlated with the concentrations of both, at 24 and 48 h of determination. For all of these compounds, the correlation coefficient was over 0.7, which indicates a strong correlation between the two parameters. The toxicity profile of compound **4c** was similar to that of indomethacin at 24 h, and it was approximately two-fold lower at 48 h. The salts **2a** and **2b** induced a significantly lower toxicity at 24 h, and at 48 h, **2b** had a similar profile to **2c,** and **2a** retained a low-toxicity tendency.

The newly synthetized compounds showed lower to high toxicity on *Daphnia magna*. Thus, pyrroles **4a** and **4d** induced, at 24 h of exposure, lethality values below 35%, whereas for pyrroles **4b** and **4c**, the lethality values were greater than 80%. Both compounds **4b** and **4c** induced 100% lethality at 48 h of exposure at all tested concentrations. For all newly synthetized compounds, low correlations between the concentrations and the effect were recorded.

The LC_50_ value of indomethacin at 48 h on *D. magna* calculated in our work was 25.12 µM, a value that is close to that predicted (15.85 µM) by Qui et al. [45]. Pyrroles **4a** and **4d** showed a lower toxicity compared with indomethacin, whereas **4b** and **4c** showed a significantly higher toxicity than the positive control.

The results of the two crustacean bioassays highlight the differences in sensitivity of the two species. Thus, *D. magna* was more sensitive to the tested compounds than *Artemia franciscana*, for which most of the tested compounds were found to be nontoxic. Compounds **2c** and **4c** showed moderate toxicity on *Artemia franciscana*, whereas on *Daphnia magna*, **4b** and **4c** showed high toxicity; **2a**, **2b**, and **2c** were moderate to highly toxic; only **4a** and **4d** were nontoxic. The high sensitivity of *Daphnia magna* towards *Artemia salina* was also observed in other studies on various chemicals such as the mixture of detergents and pesticides [46,47].

#### 2.2.3. Compound-Mediated Cytotoxicity Assays

**Figure 9:**
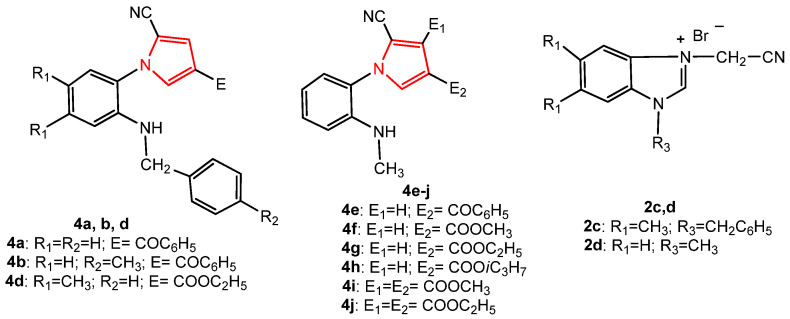
The structure of the pyrrole derivatives tested for antitumor activity.

In order to study the antitumor activity of the pyrrole derivatives, in vitro drug-mediated cytotoxicity assays were performed using tumor cell lines derived from colon (LoVo), breast (MCF-7), ovary (SK-OV-3) human adenocarcinomas as well as normal endothelial HUVECs as control. The newly synthesized compounds, **2c, 4a**, **4b**, and **4d**, and the known pyrroles, **4e**–**4j**, together with their intermediate salt, **2d** [36] (Figure 9), were tested for their potential cytotoxic activity. The structural characteristics of the known synthesized compounds, pyrroles **4e**–**4j**, and **2d** were in agreement with the data in the literature [36].

Therefore, cancer and normal control cell lines were subjected to compound-mediated cytotoxicity experiments using MTS colorimetric assays in order to assess the cytotoxic activity of the pyrroles under study and to discriminate among them regarding their ability to inhibit cell proliferation. The capacity of the pyrrole derivatives to inhibit tumor cell proliferation was compared to the cytotoxic activity of several drugs used in oncological treatments: cisplatin (Cis-Pt), frequently used in colon and ovary cancer treatment; doxorubicin (Dox), routinely used in breast cancer treatment; 5-fluorouracyl (5-FU), used in colon and breast cancer treatments. The assays were carried out on three cancer cell lines of different histological origin vs. normal human endothelial cells, used as reference, in order to modulate the chemo-sensitivity of cancer cells to drug treatments, and to find alternative therapeutic agents to the classical drugs that might overcome or reverse the chemo-resistance that usually installs after several cycles of chemotherapy.

**Figure 10:**
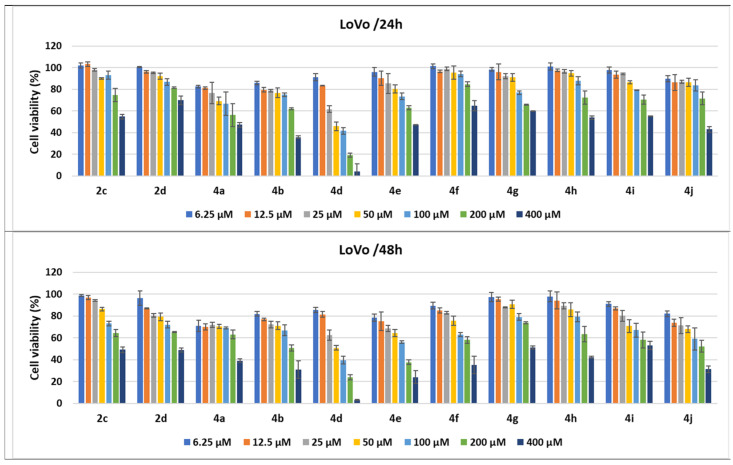
Antitumor effect of pyrrole derivatives against LoVo colon cancer cells. The viability of the LoVo cells was measured after treatments with scalar concentrations of pyrrole compounds for 24 or 48 h and compared to that of untreated control cells. Data are expressed as the mean values ± standard deviations (SDs) of three different experiments (*n = 3*).

**Figure 11:**
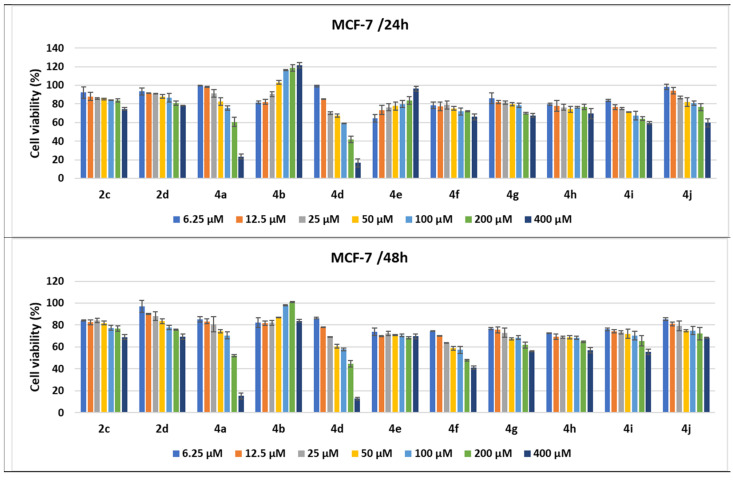
Antitumor effect of pyrrole derivatives against MCF-7 breast cancer cells. The viability of the MCF-7 cells was measured after treatments with scalar concentrations of pyrrole compounds for 24 or 48 h and compared to that of untreated control cells. Data are expressed as the mean value ± standard deviations (SDs) of three different experiments (*n = 3*).

**Figure 12:**
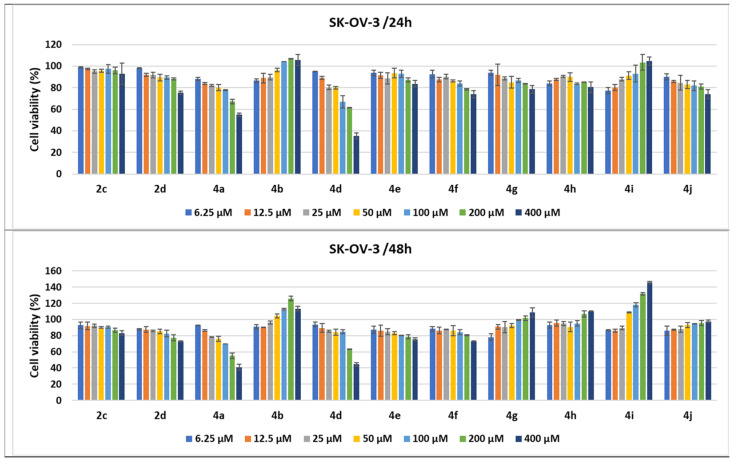
Antitumor effect of pyrrole derivatives against SK-OV-3 ovary cancer cells. The viability of the SK-OV-3 cells was measured after treatments with scalar concentrations of pyrrole compounds for 24 or 48 h and compared to that of untreated control cells. Data are expressed as the mean values ± standard deviations (SDs) of three different experiments (*n = 3*).

**Figure 13:**
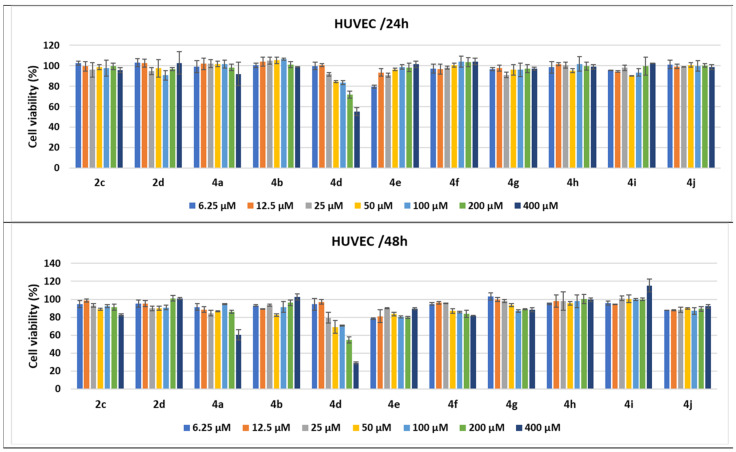
Antitumor effect of tested compounds against HUVECs. The cell viability was measured after HUVEC treatments with scalar concentrations of the tested compounds for 24 or 48 h and compared to that of untreated control cancer cells. Data are expressed as the mean values ± standard deviations (SDs) of three different experiments (*n = 3*).

**Figure 14:**
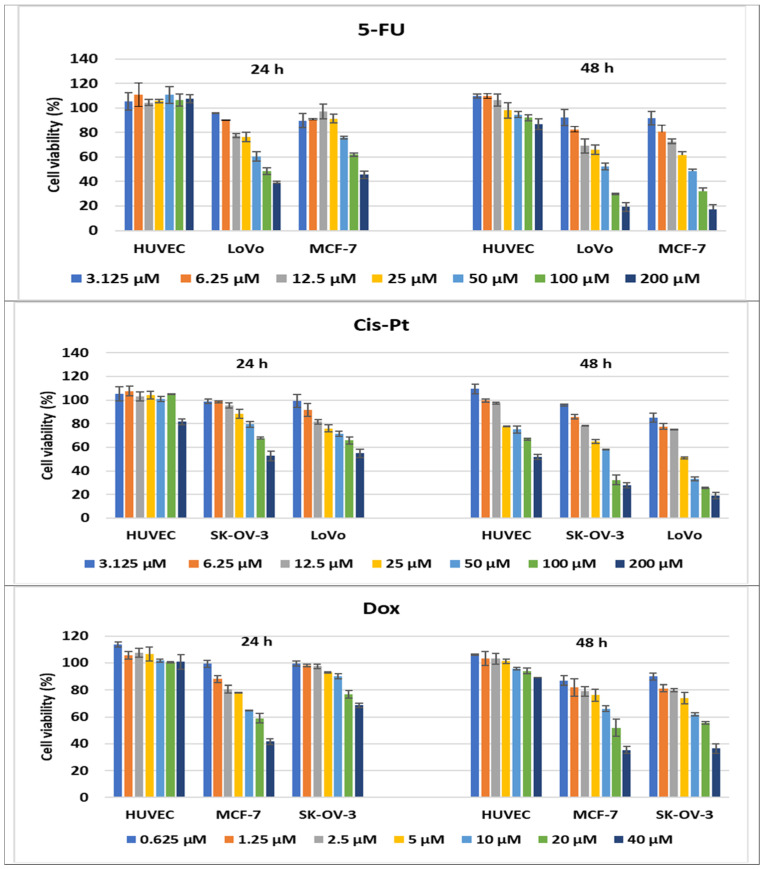
Antitumor effect of oncolitical control drugs against cancer vs. normal cell lines. The cell viability was measured after cell treatments with scalar concentrations of drugs for 24 or 48 h and compared to that of untreated control cells. Data are expressed as the mean values ± standard deviations (SDs) of three different experiments (*n =* 3).

Increasing concentrations of pyrroles, ranging from 6.25 to 400 μM, or oncological drugs (from 3.125 to 200 μMfor Cis-Pt and 5-FU and from 0.625 to 40 μM for Dox) were added for 24 h or 48 h to cancer LoVo, MCF-7, SK-OV-3, and the control HUVECs. The percentages of cell viability were calculated for each compound and cell line, and the cytotoxic effects varied depending on dose, time, and cell type as shown in Figure 10, Figure 11, Figure 12, Figure 13 and Figure 14.

When the cell responses to compound treatments were analyzed, the strongest cytotoxic dose-dependent effects of the pyrroles were observed against the LoVo colon cancer cell line. Treatments with all tested pyrroles for 24 and 48 h increased cell lysis in a dose- and time-dependent manner, demonstrated by the decrease in cell viability percentages.

Thus, all pyrrole compounds decreased LoVo cell viability to 82.54% for **4a**; 85.94% for **4b**; 89.78% for **4j**; 91.11% for **4d** when the cells were treated with 6.25 μM for 24 h. An increase in the concentration to 50 μM induced a decrease in cell viability to 45.81% for **4d** and 69.13% for **4a**; the other compounds induced a decrease in cell viability between 76.74% and 95.52%. Cell viability percentages decreased until 41.62% and 66.81% when the cells were treated with 100 μM of **4d** and **4a**, respectively, while 200 μM treatments induced decreases to 19.06% for **4d**; 56.16% for **4a**; 62.17% for **4b**; 62.87% for **4e** pyrrole compounds. The 400 μM treatments for 24 h induced the highest decreases in cell viability, under 70% for all of the pyrrole compounds, the best effect being observed for **4d** (3.93%); **4b** (35.87%); **4j** (42.97%); **4a** (47.32%); **4e** (47.37%). The compounds **4h** and **4i** as well as **2c** induced a decrease in cell viability between 50 and 60%. When the treatment time for the LoVo cells was prolonged to 48 h, the cytotoxic effects of all pyrrole compounds were amplified. Even for 100 μM, **4d** induced a decrease in cell viability to 39.6%; **4e** and **4j** to 56.06% and 59.04%, respectively, while the other compounds decreased the viability by between 60 and 80%. Treatments with 400 μM of **4a**, **4b, 4d, 4e**, **4f**, **4h**, an d**4j** and salts **2c** and **2d** induced a decrease in cells’ viability to under 50%, the strongest cytotoxicity being induced by **4e** (24.02%) and **4d** (3.1%) (Figure 10).

Among the compounds under study, **4d** induced the strongest cytotoxic effect, both at 24 (3.93%) and 48 h (3.10%) for 400 μM; the ethoxycarbonyl group linked onto the pyrrole core in the fourth position and the 2-benzylamino-4,5-dimethylphenyl moiety on the N-1 position from this heterocycle might be responsible for this better activity. The same derivative, **4d**, decreased cells’ viability to under 50%, not only for the highest concentration tested but also for the three consecutive dilutions (i.e., 200, 100, and 50 μM), both at 24 and 48 h (Figure 10).

The treatments of the MCF-7 cells with the pyrrole compounds demonstrated cytotoxic features for **4a, 4d**, **4f**–**4j**, **2d**, and **2c**, and the decrease in viability was higher when the time was prolonged to 48 h. Thus, the MCF-7 tumor breast cells were affected in a dose- and time-dependent fashion by treatments with the compounds under study, since the 400 μM treatments with the **2d**, **4f**, **4g**, **4h**, and **2c** compounds decreased the MCF-7 cells’ viability, ranging from 66 to 79%, while **4i** and **4j** diminished it to 59.57% and 59.7%, respectively. The 200 μM treatments with compound **4a** and its structural homologue **4d** decreased cell viability to 41.92% and 60.66%, respectively, while the 400 μM treatments induced a greater decrease to 16.67% for **4d** and 23.54% for **4a** (Figure 11).

When the treatment time for the MCF-7 cells was prolonged to 48 h, the cytotoxic effects of all compounds were amplified. Even for 200 μM, several compounds induced a decrease in cell viability under or around 50% (**4d** to 44.77%, **4f** to 47.86%, and **4a** to 52.07%), while **4g**, **4h**, and **4i** diminished cell viability, ranging between 61.69% and 65.49%. Treatments with 400 μM of **4a**, **4d**, and **4f** induced a decrease in cells’ viability to under 50% and to 41.22% for **4f**. As already observed in the LoVo cell line, the pyrroles **4a** (with a benzoyl group and 2-benzylaminophenyl moiety) and **4d** had the strongest antitumor effect, with the cell viability percentages being 15.13% and 12.82%, respectively, at 48 h. The **4b** compound seemed to stimulate MCF-7 cell growth when the breast cancer cell line was treated for either 24 or 48 h, while **4e** seemed to stimulate cell proliferation only at 24 h of treatment (Figure 11).

When the SK-OV-3 cells were subjected to these treatments of the derivatives, the antiproliferative effects were lower than those observed in the LoVo and MCF-7 cell lines, both at 24 and 48 h. A low antitumor effect measured through the cytotoxic activity was observed for **2d**, **4e**–**4g**, and **4j**; when cells were treated for 24 h with 400 μM, cell viability percentages decreased to 83.67% for **4e** to 73.9% for **4f**. The strongest effect was observed following **4a** and **4d** treatments with 400 μM, which decreased cell viability to 54.93% and 35.27%, respectively. The SK-OV-3 ovary cancer cells seemed not to be affected much by the **4h** and **2c** treatments, while **4i** and **4b** had an inverse effect, increasing cell viability. When the treatment time was prolonged to 48 h, cell viability percentages slightly diminished following **2d**, **4e**, **4f**, and **2c** treatments, compared to 24 h of incubation. The effect was amplified by the prolonged treatment at 48 h for **4a**, diminishing cell viability to 40.84%. In contrast, 48 h treatments with the **4g**, **4h**, and **4j** pyrroles seemed to have an inverse effect, while **4i** and **4b** clearly induced a dose-dependent proliferation of the ovary cancer cells (Figure 12).

In addition to the experiments performed on cancer cell lines derived from various human solid adenocarcinomas, the same 24 and 48 h treatments were applied on normal HUVECs, used as control cells in the tests. Treatment of HUVECs for 24 h with scalar concentrations of the tested compounds had no influence on cell growth or demonstrated low cytotoxicity, except the pyrrole **4d**; when cells were treated with 200 μM, the cell viability percentages diminished to 71.8%, while 400 μM induced a greater decrease to 55.10%. When the treatment time was prolonged to 48 h, several compounds, such as **4e** and **4j**, seemed to induce higher levels of cell lysis, over 10%, while **4f**, **4g**, and **2c** diminished cell viability to under 90% when used at high concentrations. Concentrations higher than 25 μM of **4d** diminished cell viability to under 80%, the strongest cytotoxic effect being induced by 400 μM to 29% of cell viability. The homologue **4a** demonstrated high cytotoxic activity only for the highest concentration, decreasing cell viability to 60.41% (Figure 13).

In addition, cancer cells lines were treated with specific drugs, currently used in oncological clinics, which served as a positive control of the assays during our experiments. Higher cytotoxicity effects were observed in comparison with treatments of normal cells, and the percentages of cell viability decreased with the increase in drug concentration. Therefore, the cytotoxic activity of the compounds was compared to drug oncolytic capacity, and **4d** demonstrated the strongest cytotoxic effects on all cancer lines tested. When LoVo cells were treated with 200 μM of **4d** for 24 h, the compound demonstrated a better activity than the reference drugs, 5-FU and Cis-Pt, used at the same concentration, with 81% of the cells being killed (19% cell viability). After 48 h treatments with 200 μM of 5-FU and Cis-Pt, the cell viability percentages decreased to 19.25% and 19.08%, respectively, slightly lower than the cytotoxic effects of **4d** (23.82%) (Figure 10 and Figure 14).

Treatments of MCF-7 cells with 200 μM of 5-FU or 40 μM of Dox demonstrated comparable effects with 200 μM of **4d**, around 40% of cell viability being achieved. After 48 h of drug treatment, the cell viability was decreased more by 200 μM of 5-FU and 40 μM of Dox(17.27% and 35.41%, respectively) compared to **4d**, which induced a decrease to 44.77% in the MCF-7 cell viability percentages; on the other hand, the treatment with 400 μM of **4d** decreased cell viability to 16.67% and 12.82% after 24 and 48 h, respectively (Figure 11 and Figure 14).

Ovary cancer cells are known for their drug resistance to chemotherapeutic treatments, but 200 μM of **4a** and **4d** had comparable effects with 40 μM of Dox, and a slightly lower effect than 200 μM of Cis-Pt when treated for 24 h. The increase in the incubation time of the SK-OV-3 cells with Dox and Cis-Pt diminished the cell viability more than the compounds, to 36.42% and 27.77%, respectively, for the 200 μM concentration of Cis-Pt and 40 μM of Dox (Figure 12 and Figure 14).

The 24 h treatments of HUVECs with the conventional drugs had no cytotoxic effects, except the 200 μM treatment with Cis-Pt, which induced a decrease in cell viability to 81.58%. When the incubation time increased to 48 h, the 200 μM treatment with 5-FU and 40 μM of Dox diminished the percentages of cell viability to 86.63% and 89%, respectively. Moreover, Cis-Pt induced a decrease in cell viability between 77.59% and 51.80% when used at concentrations ranging from 25 to 200 μM (Figure 14).

### 2.3. Prediction of the Molecular Mechanism of Action

Using 2D structural descriptors, the PASS application was used to predict the probable mechanisms of action for the compounds **4a**–**j**. Sunitinib was used as a comparative. The results were analyzed if the probability of each compound being active (Pa) was higher than the probability of being inactive (Pi) on each target [48]. The analysis identified seven effects that were directly connected to the antitumor action, and they are presented in Table 3.

The Pa values indicated the most probable biological effects and targets but not the potency of that effect [49]. The results indicated that the new compounds, **4a**–**d**, are potential inhibitors of PDGFR kinase and, consecutively, produce the inhibition of angiogenesis process. Another predicted mechanism involves the focal adhesion kinases (FAKs). FAKs are overexpressed in many tumor cells and promote tumor angiogenesis and vascular permeability, being an attractive targets for anticancer therapies [50]. The compounds **4e**–**j** were also predicted to inhibit the angiogenesis process but targeting only FAKs and not PDGFR. The difference might be caused by the presence of the additional benzene ring present in compounds **4a**–**d**.

A series of medicinal chemistry measures, ADME, and toxicity endpoints were estimated using the ADMETLab2.0 platform [51]. The data are presented in Table 4.

The new compounds **4a**–**d** were designed to respect the Lipinski rule and were not predicted to be pan assay interference compounds (PAINS). A medium risk of hepatic toxicity was predicted but no skin or respiratory toxicity. The compounds **4a** and **4b** were predicted to have no mutagenic or carcinogenic effects, but for their analogs **4c** and **4d**, a medium risk was estimated.

## 3. Materials and Methods

### 3.1. Chemistry

All chemicals were of analytical grade and were purchased from common commercial source (Merck, Sigma-Aldrich, and Alfa Aesar). The NMR spectra were registered on a Varian Gemini 300BB spectrometer (Varian, Palo Alto, CA, USA) operating at 300 MHz for ^1^H and 75 MHz for ^13^C, at 298 K, using TMS as the internal standard and CDCl_3_ or DMSO-d_6_ as solvents (Supplementary Materials). The chemical shifts *δ* are reported in parts per million (ppm) and the coupling constants values *J* are in hertz (Hz). The splitting patterns are abbreviated as follows: s, singlet; d, doublet; dd, doublet of doublets; t, triplet; m, multiplet; b, broad. The FT-IR spectra were measured on a Vertex 70 spectrometer (Bruker Optik GmbH, Ettlingen, Germany) in ATR modes (Supplementary Materials). The X-ray diffraction measurements were registered with a Rigaku Oxford-Diffraction XCALIBUR E CCD diffractometer (Rigaku Oxford Diffraction, Sevenoaks, Kent, UK) equipped with MoKα radiation. Single crystals were positioned at 40 mm from the detector and 665 frames and were measured each for 30 s over 1° oscillation width. The data integration were carried out using the CrysAlis program [52]. The structure was solved by Intrinsic Phasing using Olex2 [53] software with the SHELXT program [54] and refined by full-matrix least-squares on *F*^2^ with SHELXL-2015 [55] using an anisotropic model for nonhydrogen atoms. The H atoms were introduced in idealized positions using riding model. The melting points, m.p., were determined on a Boëtius hot plate microscope (Carl Zeiss, Jena, Germany) and are uncorrected. The elemental analysis was carried out on a Costech Instruments EAS 32 apparatus (Costech Analytical Technologies, Valencia, CA, USA).

#### 3.1.1. Synthesis of 1-Benzyl-5,6-Dimethyl-3-Cyanomethylbenzimidazolium Bromide **2c**

A mixture of 1-benzyl-5,6-dimethylbenzimidazole **1c** (3.54 g, 15 mmol) and bromoacetonitrile (22.5 mmol, 1.6 mL) were dissolved in acetone (150 mL). The reaction mixture was refluxed for 10 h, and the precipitate obtained after cooling was filtered off and washed with acetone and dried. The compound was obtained as colorless crystals and used for the synthesis of the new pyrroles without purification. m.p. 193–195 °C; yield 75%; Anal. Calcd. for C_18_H_18_BrN_3_ (356.26 g/mol): C, 60.68; H, 5.09; N, 11.79 Found C, 60.44; H, 5.28; N, 11.65. IR (ATR solid, cm^−1^): 2254 (ν_CN_), 3107 (ν_CH_); ^1^H-NMR (300 MHz, DMSO-d_6_) δ ppm: 2.38, 2.50 (2s, 6H, 2CH_3_), 5.80, 5.97 (2s, 4H, 2CH_2_), 7.39–7.55 (m, 5H, Ph), 7.83, 7.92 (2s, 2H, Ar), 10.02 (s, 1H, H2-pyrrole); ^13^C-NMR (75 MHz, DMSO-d_6_) δ ppm: 19.9, 20.0 (2CH_3_), 35.0 (**C**H_2_CN), 49.9 (**C**H_2_Ph), 112.8, 113.6 (C-4, C-7), 114.2 (CN), 128.3, 128.7, 128.9 (5C, Ph, tertiary), 129.0, 129.1, 133.6, 136.9, 137.1 (5C, quaternary), 142.3 (C-2).

The salts **2a** and **2b** were synthetized according to the data in the literature, and its characterization is consistent with these [34].

#### 3.1.2. General Procedure for the Synthesis of Pyrroles **4a**–**d**

A mixture of benzimidazolium bromide **2a**–**c** (3 mmol) and the acetylenic dipolarophile (3.8 mmol) in 1,2-epoxybutane (22.5 mL) was refluxed with stirring for ca. 48 h when a precipitate was formed. The solvent was evaporated under reduced pressure, and the residue was purified by column chromatography (Merck alumina (70–230 mesh), CH_2_Cl_2_) and then by crystallization from alcohol with obtaining of colorless crystals.

4-Benzoyl-2-cyano-1-(2-benzylamino-phenyl)pyrrole (**4a**)

M.p. 154–155 °C (ethanol); Yield 60%. Anal. Calcd. for C_25_H_19_N_3_O (377.44 g/mol): C, 79.55; H, 5.07; N, 11.13. Found C, 79.50; H, 5.25; N, 13.33. IR (ATR solid, cm^−1^): 1632 (ν_C=O_), 2217 (ν_C≡N_), 3120 (ν_CH_), 3358 (ν_NH_). ^1^H-NMR (300 MHz, CDCl_3_) δ ppm: 3.85 (bs, 1H, NH), 4.30 (d, 2H, *J* = 3.0 Hz, CH_2_), 6.70–6.77 (m, 2H, H-3’, H-5’, *N*-aryl), 7.12 (dd, 1H, *J* = 2.6, 1.6 Hz, H-6’, *N*-aryl), 7.19–7.28 (m, 6H, PhCH_2_, H-4’, *N*-aryl), 7.38 (d, 1H, *J* = 1.7 Hz, H-3, pyrrole), 7.40–7.44 (m, 2H, *meta*- PhCO), 7.46 (d, 1H, *J* = 1.7 Hz, H-5, Pyrrole), 7.49–7.55 (m, 1H, *para*-PhCO), 7.74–7.78 (m, 2H, *ortho*-PhCO); ^13^C-NMR (75 MHz, CDCl_3_) δ ppm: 47.6 (CH_2_), 107.5, 122.6 (2C, quaternary), 111.8 (C-3’), 112.7 (CN), 117.4 (C-5’), 122.4 (C-3), 125.7 (C-4), 127.8 (C-6’), 127.1, 127.5, 128.5, 138.1 (6C, CH_2_Ph), 132.4 (C-4’), 132.6 (C-5), 128.8, 128.9, 131.5, 138.3 (6C, Ph), 143.5 (C-1’), 188.8 (CO).

4-Benzoyl-2-cyano-1-[2-(4-methylbenzylamino)phenyl)]pyrrole (**4b**)

M.p. 127–129 °C (2-propanol); Yield 45 %. Anal. Calcd. for C_26_H_21_N_3_O (391.46 g/mol): C, 79.77; H, 5.41; N, 10.73. Found C, 80.12; H, 6.05; N, 11.12. IR (ATR solid, cm^−1^): 1636 (ν_C=O_), 2301 (ν_C≡N_), 3125 (ν_CH_), 3362 (ν_NH_). ^1^H-NMR (300 MHz, CDCl_3_) δ ppm: 2.32 (s, 3H, CH_3_), 3.85 (t, 1H, *J* = 5.2 Hz, NH), 4.30 (d, 2H, *J* = 5.2 Hz, CH_2_), 6.77–6.82 (m, 2H, H-3’, H-5’, *N*-aryl), 7.12–7.25 (m, 5H, PhCH_2_, H-6’, *N*-aryl), 7.28–7.34 (m, 1H, H-4’, *N*-aryl), 7.44–7.52 (m, 4H, H-3, H-5, *meta*-PhCO), 7.56–7.58 (m, 1H, *para*-PhCO), 7.81–7.84 (m, 2H, *ortho*-PhCO); ^13^C-NMR (75 MHz, CDCl_3_) δ ppm: 21.0 (CH_3_), 47.6 (CH_2_), 107.6, 122.6 (2C, quaternary), 112.0 (CN), 112.8 (C-3’), 117.4 (C-5’), 122.5 (C-3), 125.8 (C-4), 127.2 (C-6’), 127.9, 128.6, 135.1, 137.3 (6C, C_6_H_4_), 129.0, 129.6, 131.5, 138.4 (6C, Ph), 132.5 (C-4’), 132.8 (C-5), 143.6 (C-1’), 188.9 (CO).

Methyl 1- [(2-benzylamino-4,5-dimethyl)phenyl]-2-cyanopyrrole-4-carboxylate **(4c)**

M.p. 101–102 °C (ethanol); Yield 41 %. Anal. Calcd. for C_22_H_21_N_3_O_2_ (359.42 g/mol): C, 73.52; H, 5.89; N, 11.69; Found C, 73.84; H,6.12; N, 11.91. IR (ATR solid, cm^−1^): 1708 (ν_CO_), 2216 (ν_CN_), 3131 (ν_CH_), 3427 (ν_NH_). ^1^H-NMR (300 MHz, CDCl_3_) δ ppm: 2.16, 2.21 (2s, 6H, 2CH_3_), 3.83 (s, 3H, CH_3_O), 4.32 (s, 2H, CH_2_Ph), 6.60, 6.90 (2s, 2H, *N*-aryl), 7.31–7.47 (m, 7H, 2H-Pyrrole, 5H-Ph); ^13^C-NMR (75 MHz, CDCl_3_) δ ppm: 18.6, 20.3 (2CH_3_), 48.02 (CH_2_Ph), 51.8 (**C**H_3_O), 107.3 (C-4), 112.2 (CN), 118.0 (C-2), 120.6, 125.7, 138.7, 140.2, 141.4 (5C, quaternary), 121.6 (C-3), 131.9 (C-5), 114.2, 127.2, 127.4, 128.5, 128.8 (7C, tertiary), 163.2 (COO).

Ethyl 1- [(2-benzylamino-4,5-dimethyl)phenyl]-2-cyanopyrrole-4-carboxylate (**4d**)

M.p. 115–117 °C (ethanol); Yield 35%. Anal. Calcd. for C_23_H_23_N_3_O_2_ (373.45 g/mol): C, 73.97; H, 6.21; N, 11.25; Found C, 74.22; H, 6.57; N, 11.46. IR (ATR solid, cm^−1^): 1706 cm^−1^ (ν_CO_), 2222 (ν_CN_), 3136 (ν_CH_), 3387 (ν_NH_). ^1^H-NMR (300 MHz, CDCl_3_) δ ppm: 1.33 (t, 3H, *J* = 7.1 Hz, CH_2_-**CH_3_**), 2.16, 2.21 (2s, 6H, 2CH_3_), 3.60 (bs, 1H, NH), 4.26–4.33 (m, 4H, CH_2_Ph, CH_2_O), 6.57, 6.89 (2s, 2H, *N*-aryl), 7.25–7.32 (m, 5H, Ph), 7.33 (d, 1H, *J* = 1.7 Hz, H-3), 7.47 (d, 1H, *J* = 1.7 Hz, H-5); ^13^C-NMR (75 MHz, CDCl_3_) δ ppm: 14.5 (CH_2_-**C**H_3_), 18.6, 20.3 (2**C**H_3_), 48.0 (CH_2_Ph), 60.6 (**C**H_2_O), 107.3 (C-4), 112.2 (CN), 118.4 (C-2), 120.6, 125.6, 138.7, 140.1, 141.4 (5C, quaternary), 121.6 (C-3), 131.8 (C-5), 114.2, 127.2, 127.4, 128.5, 128.8 (7C, tertiary), 162.8 (COO).

### 3.2. Toxicity Evaluation

#### 3.2.1. Phytotoxicity Evaluation

The Triticum bioassay was used to evaluate the toxicity of the compounds on plant cells. The method consisted in tracking the elongation of the root at certain time intervals in the presence of established concentrations of the compounds of interest [56,57]. Wheat seeds of similar size were used. Seeds featuring 1 cm roots were selected from the germinated seeds placed in Linhart pots in optimal conditions (humidity, temperature of 25 and darkness). 11 such seeds were placed in 10 cm sized Petri dishes. Solutions of the compounds of interest were put in these vessels in advance. Indomethacin was chosen as control, because it contains a pyrrole core (indole) and it demonstrated anticancer through inhibition of VEGF. [Wang HM, Zhang GY. Experimental study of the inhibitory effect of indomethacin on the growth and angiogenesis of human colon cancer xenografts [58]. For each compound, 5 dilutions were made between 10 and 1000 μM. Two controls were used: indomethacin diluted in the same manner as the tested compounds, and 1% DMSO water solution. The root elongation was measured over 3 days as follows: at 24, 48, and 72 h. The microscopic analysis was performed after 24 h of contact with the test solutions on one of the 11 seeds. 0.5 cm root tips were stained with acetic orcein [59]. The appearance of the nucleus, cytoplasm, cell wall, and changes in mitotic film were tracked. A clear field microscope was used. Euromex oxion series 110–240 V/50–60 Hz with digital camera CEMEX 5 DC 5000C and 40× and 100× lenses (for this we used cedar oil immersion) (Sigma-Aldrich St. Louis, MO, USA).

#### 3.2.2. Animal Toxicity Assay

##### *Artemia franciscana* Toxicity Assay

The sea shrimp lethality test was conducted on *Artemia franciscana* Kellog. The shrimp, sourced from the Great Salt Lake, were procured from a commercial supplier (S.K. Trading, Thailand, which repackaged them from Ocean Star International, London, UK). Artificial seawater was prepared by dissolving a commercially sourced pre-blended salt mix (Coral Marine, Grotech, Hunt Valley, MD, USA) in distilled water using ultrasound at a concentration of 33.5 g/L. The hatching process was initiated 48 h prior to the onset of the test, at ca. 25 °C, under strong aeration. The test was carried out in 24-well plates (6 × 4), with suspensions of test compounds placed in triplicate in the wells. The following concentrations were used: 60, 125, 250, 500, and 1000 (μM). Artificial seawater was used as a negative control. After hatching, 10–20 nauplii were transferred to each well using a 100 μL pipette. In addition, 24 and 48 h after introduction into the test suspensions, all live and dead nauplii in each well were counted.

##### *Daphnia magna* Toxicity Assay

*Daphnia magna* Straus young were selected from a culture maintained parthenogenetically at 25 °C, and a photoperiod of 16 h/8 h light/dark cycle in a Sanyo MLR-351H climatic chamber (Sanyo, San Diego, CA, USA). Each compound was tested on ten daphnids in six concentrations ranging from 12.5 to 500 µM, using two replicates. The bioassay was performed in 12 well tissue culture plates (Greiner Bio-One, Kremsmünster, Austria) [60,61,62,63]. Indomethacin was tested at the same concentrations as the samples, and a 1% DMSO solution was used as a negative control. The concentrations were selected based on the solubility and a pre-screening assay. For each sample, the lethality value was recorded at 24 and 48 h of exposure. LC_50_ values and the 95% confidence intervals (95%CI) for LC_50_ values were calculated using the least square fit method.

### 3.3. Prediction of the Molecular Mechanism of Action

The SMILES codes of the target compounds were introduced as in the application PASS (Prediction of Activity Spectra for Substances) to evaluate the potential interactions with a large number of biological relevant molecules. Each compound’s effects and target interactions profile was manually analyzed in order to select the relevant oncotargets.

### 3.4. Statistical Analyses

For phytotoxicity and Artemia franciscana toxicity assessment, statistical analyses were performed in R, v. 4.1.3 [64], under Rstudio, v. 2022.02.0, Build 443 [65]. For phytotoxicity a robust mixed-effects model was used (R package “robustlmm”) [66], with compounds and concentration treated as fixed factors, and day of measurement treated with a fixed and a random component. *p*-Values (which are rather controversial in the case of mixed-effects models) were estimated based on Kenward–Roger approximated degrees of freedom, as computed by the “sjPlot” R package [67]. Interaction effects plots were also generated with the help of the “sjPlot” package.

To estimate LC_50_ for *Artemia franciscana* toxicity, three-parameter Weibull functions were used, as implemented in the R “drc” package [68], the models being selected among several possible options based on the log likelihood value and Akaike’s information criterion.

### 3.5. Cell Cytotoxicity

#### 3.5.1. Cell Cultures and Treatments

The potential cytotoxic activity of the pyrrole derivatives under study was evaluated in three standardized adherent human cancer cell lines vs. normal human endothelial cells and compared with the cytotoxicity of oncolitic drugs routinely used for cancer treatments. The MCF-7 human Caucasian breast adenocarcinoma and SK-OV-3 human Caucasian ovary adenocarcinoma cell lines were purchased from European Collection of Authenticated Cell Cultures (ECACC), while LoVo human colorectal adenocarcinoma cancer cell line, and HUVEC human umbilical vein endothelial cells were purchased from American Type Culture Collection (ATCC).

The stock solutions of pyrrole derivatives were prepared by dissolving them in a minimum amount of DMSO, and preserved at 4 °C. The control drugs Cisplatin (Cis-diammineplatinum(II) dichloride, DDP), and Doxorubicin were purchased from Sigma Aldrich (St. Louis, MO, USA), and stock solutions of 5 mM were prepared as recommended, in sterile distilled water, and preserved at –20 °C; the stock solution of 5 mM of 5-Fluorouracyl (Sigma Aldrich) was prepared in absolute ethanol, and preserved at 4 °C. All working solutions were prepared from the stocks by serial dilutions in culture medium before each experiment.

Adherent normal and cancer cells were routinely cultured in DMEM/F12 medium added with 10% fetal bovine serum, 2 mM L-glutamine, 100 units/mL penicillin, 100 µg/mL streptomycin (Sigma Aldrich, St. Louis, MI, USA) in culture flasks, and incubated at 37 °C in 5% CO_2_ humidified atmosphere [2,69]. When cultured cells achieved around 60% confluence, they were detached from flasks with a nonenzymatic solution of PBS/1 mM EDTA, washed twice in PBS and immediately used for assessing the cell viability in a colorimetric cytotoxicity assay. Non-treated cells were used as controls throughout all experiments [2,70].

#### 3.5.2. MTS Cytotoxicity Assay

For the assessment of drug-induced cytotoxicity, the CellTiter 96 Aqueous One Solution Cell Proliferation Assay (Promega, Madison, WI, USA), a MTS (3-(4,5-dimethylthiazol-2-yl)-5-(3-carboxymethoxy-phenyl)-2-(4-sulfophenyl)-2H-tetrazolium, inner salt) colorimetric assay was used. The method is based on the capacity of metabolically active cells to reduce the yellow tetrazolium salt MTS to the coloured formazan, a compound that is soluble in culture medium.

All the experiments were performed in triplicate in 96-well flat bottom microtiter plates (Falcon, Teterboro, NJ, USA). Briefly, 1.5 × 10^4^ cancer or normal cells/well were cultured in 100 μL for 24 h, the culture supernatants were discarded, and cells were treated for additional 24 h or 48 h with increasing concentrations of pyrrole derivatives or oncolytic drugs. After incubation, 20 μL of colouring mixture reagent (MTS and PES (phenazine methosulfate), the last having a high chemical stability, and could combine with MTS to form a stable solution) were added in each well, and then the plates were incubated at 37 °C for 4 h, with mild agitation every 20 min. The colour developed during incubation, and it was spectrophotometrically quantified at λ = 492 nm by using a Dynex ELISA reader (DYNEX Technologies–MRS, Chantilly, VA, USA) [2,69,71].

The percentages of cell viability of the treated cells were calculated and compared to the untreated cells (considered 100% viable):

Cell viability (%) = (T − B)/(U − B) × 100

where, T = absorbance of treated cells, U = absorbance of untreated cells, and B = absorbance of culture medium (blank), for λ = 492 nm

Cell lysis (%) = 100% − Cell viability (%)

The cell viability data were expressed as the mean values ± standard deviations (SD) of three different experiments. In addition, a MTS assay was performed in the absence of cells, all the concentrations of the compounds under study being tested for their potential interference with MTS reagents, and absorbance values were extracted during calculations. Moreover, a simultaneous assay was performed in the same experimental conditions for the evaluation of DMSO potential cytotoxicity, using serial dilutions of the reagent. The lack of cellular cytotoxicity was observed at lower concentrations than 1% DMSO (data not shown).

#### 3.5.3. Statistical Analysis

All assays were performed in triplicate. Data analyzed by using Student’s paired *t* test with *p* < 0.05 were considered statistically significant.

## 4. Conclusions

In this work, we synthesized new compounds from pyrrole class through the reaction of some benzimidazolium bromide derivatives with asymmetric acetylenic dipolarophiles, implied the 1,3-dipolar cycloaddition, in 1,2-epoxybutane medium. The benzimidazolium bromide intermediates have been obtained by the reaction of some benzimidazole derivatives with bromoacetonitrile. The structure of the newly obtained compounds was elucidated by spectral data (IR, NMR) and X-ray single-crystal diffraction in case of representative pyrrole. The cytotoxicity of the tested compounds was assessed on *Triticum aestivum* L. *root* and on *Artemia franciscana* Kellogg and *Daphnia magna* crustacean, and in vitro on three human cancer cell lines (i.e., LoVo, SK-OV-3, and MCF-7) of different histologic origin. Both crustacean assays indicated that **4a** and **4d** were nontoxic, and that **2c** and **4c** had a moderate to high toxicity. In addition, *D. magna* was also sensitive to **2a**, **2b**, and **4b**, which exert moderate to high toxicity. From the tested compounds, several pyrrole derivatives displayed satisfactory anticancer activities against LoVo, SK-OV-3, MCF-7 cells, and very low cytotoxic effects towards the normal HUVECs. The in vitro compound-mediated cytotoxicity assays demonstrated dose- and time-dependent cytotoxic activity for several pyrrole compounds, the highest antitumor properties being assessed for **4a** and its homologue **4d**, especially against colon cancer cells. The obtained results prompted us to further expand our studies in order to improve the anticancer properties and better-biological activity of the pyrrole derivatives described above, to reduce the undesired side-effects, and develop a new class of promising therapeutic agents.

## Figures and Tables

**Table 1:** Crystal data and details of the data collection for **4d**.

Empirical Formula	C_23_H_23_N_3_O_2_
Fw	373.44
Space group	*P*-1
*a* (Å)	8.0707(3)
*b* (Å)	16.1583(6)
*c* (Å)	17.0040(6)
α (°)	73.404(3)
β (°)	103.491(3)
γ (°)	90.540(3)
*V* (Å^3^)	2062.55(13)
*Z*	4
*r*_calcd_ (g cm^−3^)	1.203
Crystal size (mm)	0.20 × 0.10 × 0.04
T (K)	293
*μ* (mm^−1^)	0.078
2Θ range (°)	4.188 to 58.826
Reflections collected	30622
Independent reflections	9845 (*R*_int_ = 0.0443)
Data/restraints/parameters	9845/73/494
*R*_1_ (a)	0.0738
*wR*_2_ (b)	0.2087
GOF (c)	1.000
Largest difference in peak/hole (e·Å^−3^)	0.23/−0.29
CCDC No.	2166919

**Table 2:** Daphnia magna bioassay.

Compound	24 h			48 h		
	Maximum L% (%)	LC50_50_ (µM)	95% CI of LC_50_ (µM)	Maximum L% (µM)	LC_50_ (%)	95% CI of LC_50_ (µM)
**2a**	55	471.7	424.8–523.7	100	77.51	40.44–148.6
**2b**	75	396.9	359.0–438.9	100	163.1	139.0–191.4
**2c**	100	157	135.2–182.3	100	53.59	45.33–63.34
**4a**	35	ND *	ND *	35	ND *	ND *
**4b**	85	ND **	ND **	100	ND **	ND **
**4c**	100	4.583	0.1717–122.3	100	ND **	ND **
**4d**	15	0.4	ND *	60	ND *	ND *
Indomethacin	100	128.6	112.9–146.5	100	25.12	18.01–35.03

LC_50_—lethal concentration that killed 50% of the crustaceans; 95% CI—the 95% confidence interval of the LC_50_; ND—not determined; *—LC_50_ could not be calculated because the maximum L% was below 50%; **—LC_50_ could not be calculated because the maximum L% was between 85% and 100%.

**Table 3:** The PASS predicted probabilities of the new pyrrole derivatives to be active (Pa) towards oncological proteins. Sunitinib (Su) was used as a comparative.

Target/Effect	2c	2d	4a	4b	4c	4d	4e	4f	4g	4h	4i	4j	Su
Angiogenesis inhibitor	-	-	0.45	0.41	0.37	0.32	0.36	0.31	0.24	0.25	0.36	0.29	0.84
Colony-stimulating factor antagonist	-	-	0.51	0.48	0.40	0.37	0.53	0.46	0.41	0.40	0.45	0.41	-
Focal adhesion kinase 2 inhibitor	-	0.24	0.26	0.25	0.28	0.25	0.31	0.36	0.33	0.19	0.39	0.36	-
Focal adhesion kinase inhibitor	-	-	0.19	0.18	0.21	0.18	0.20	0.24	0.20	-	0.27	0.22	-
Kinase inhibitor	-	-	-	0.25	0.29	-	0.24	0.33	-	-	-	-	0.83
Platelet-derived growth factor receptor kinase inhibitor	-	-	0.34	0.37	0.32	0.35	-	-	-	-	-	-	0.86
Proto-oncogene tyrosine-protein kinase Fgr inhibitor	-	0.18	0.29	0.30	0.28	0.25	0.36	0.34	0.31	0.29	0.35	0.33	-

**Table 4:** ADMET predictions for the new compounds.

Property	4a	4b	4c	4d
Lipinski rule	yes	yes	yes	yes
PAINS	no	no	no	no
Human intestinal absorption	<30%	<30%	<30%	<30%
Human hepatotoxicity	++	++	++	++
Drug-induced liver injury	+++	+++	+	+
AMES toxicity	++	++	−−	−−−
Carcinogenicity	+	+	−−−	−−−
Skin sensitization	−−	−−−	−	−−
Respiratory toxicity	−−−	−−−	−−−	−−−

## Data Availability

The data presented in this study are available upon request from the corresponding authors.

## Supplementary Materials

The following supporting information can be downloaded at: https://www.mdpi.com/article/10.3390/ijms23168854/s1.
