# Supplementary material for: New Pyrrole Derivatives as Promising Biological Agents: Design, Synthesis, Characterization, In Silico, and Cytotoxicity Evaluation
Source: Int J Mol Sci. 2022 Aug 9;23(16):8854. doi: 10.3390/ijms23168854 (PMC9408590; doi:10.3390/ijms23168854)
Supplement: Supplementary file 1 [file ijms-23-08854-s001.zip › ijms-1836304-supplementary.pdf]

New pyrroles derivatives as promising biological agents: Design, Synthesis, Characterization, *In Silico* and Cytotoxicity Evaluation

Beatrice-Cristina Ivan[1], Stefania-Felicia Barbuceanu[1,*], Camelia Mia Hotnog[2], Adriana Iuliana Anghel[1], Robert Viorel Ancuceanu[1], Mirela Antonela Mihaila[2], Lorelei Irina Brasoveanu[2], Sergiu Shova[3], Constantin Draghici[4], Octavian Tudorel Olaru[1,*], George Mihai Nitulescu[1], Mihaela Dinu[1], and Florea Dumitrascu[4]

[1] Faculty of Pharmacy, “Carol Davila” University of Medicine and Pharmacy, 6 Traian Vuia street, 020956 Bucharest, Romania

[2] Center of Immunology, “Stefan S. Nicolau” Institute of Virology, Romanian Academy, 285 Mihai Bravu Ave., 030304 Bucharest, Romania

[3] Laboratory of Inorganic Polymers, “Petru Poni” Institute of Macromolecular Chemistry, Aleea Grigore Ghica Voda, 41A, 700487 Iasi, Romania

[4] “C.D. Nenitescu” Institute of Organic and Supramolecular Chemistry, Romanian Academy, 202B Splaiul Independenței, 060023 Bucharest, Romania

* Correspondence: stefania.barbuceanu@umfcd.ro (S.-F.B.); octavian.olaru@umfcd.ro (O.T.O.)

Supplementary Material

1. The IR spectra

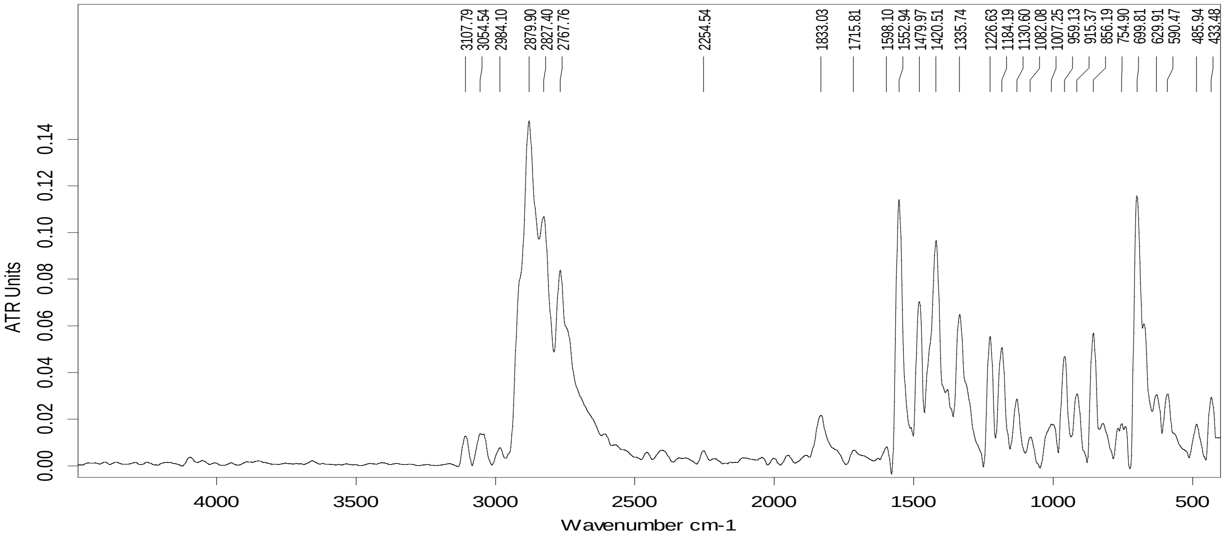

Figure S1. The IR spectrum of salt **2c**

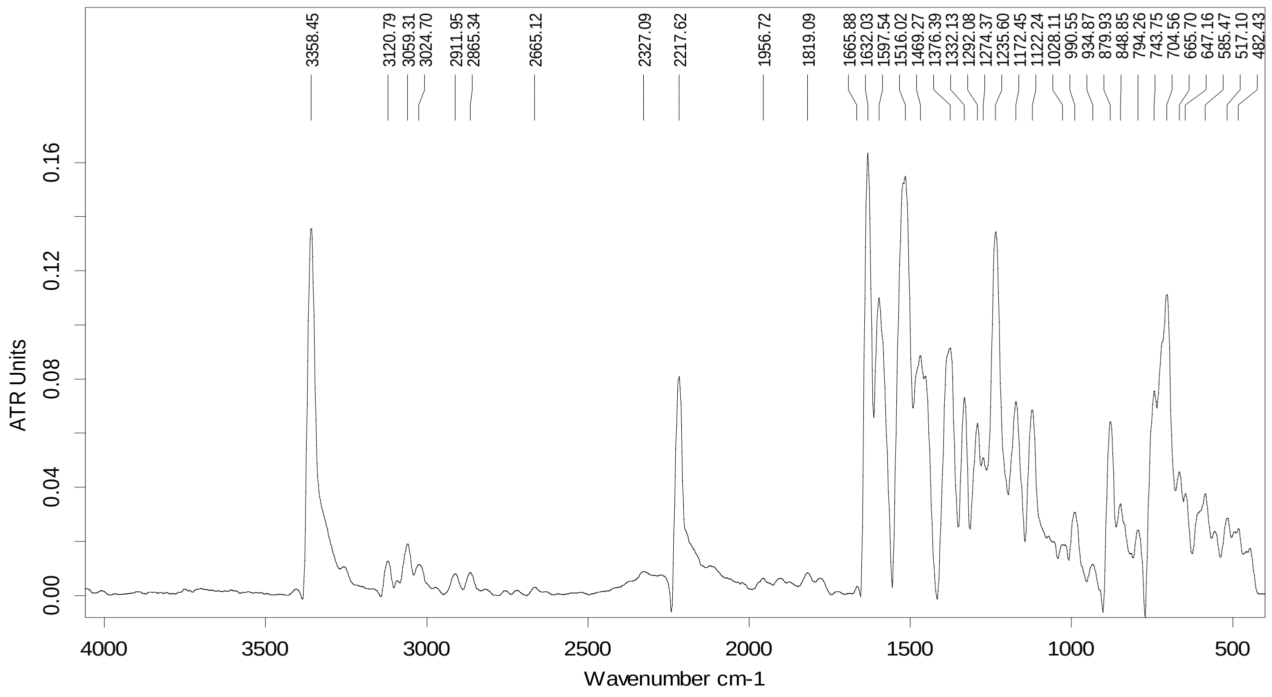

Figure S2. The IR spectrum of pyrrole **4a**

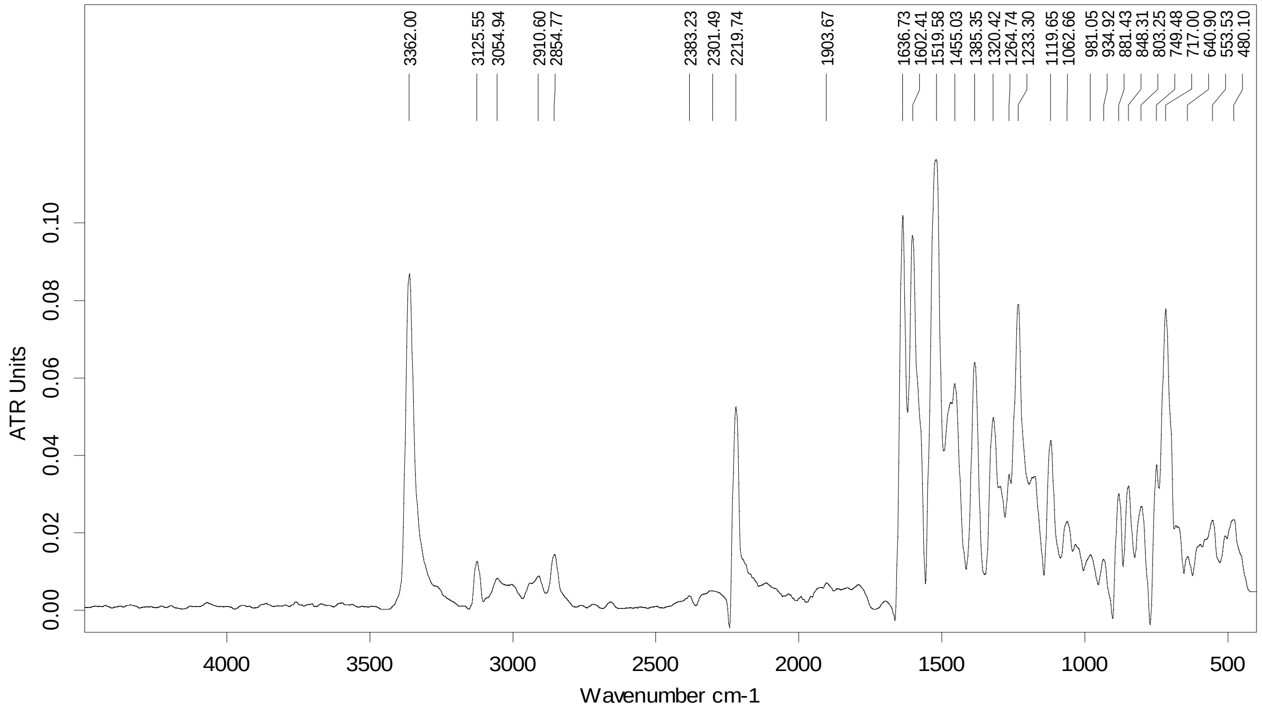

Figure S3. The IR spectrum of pyrrole **4b**

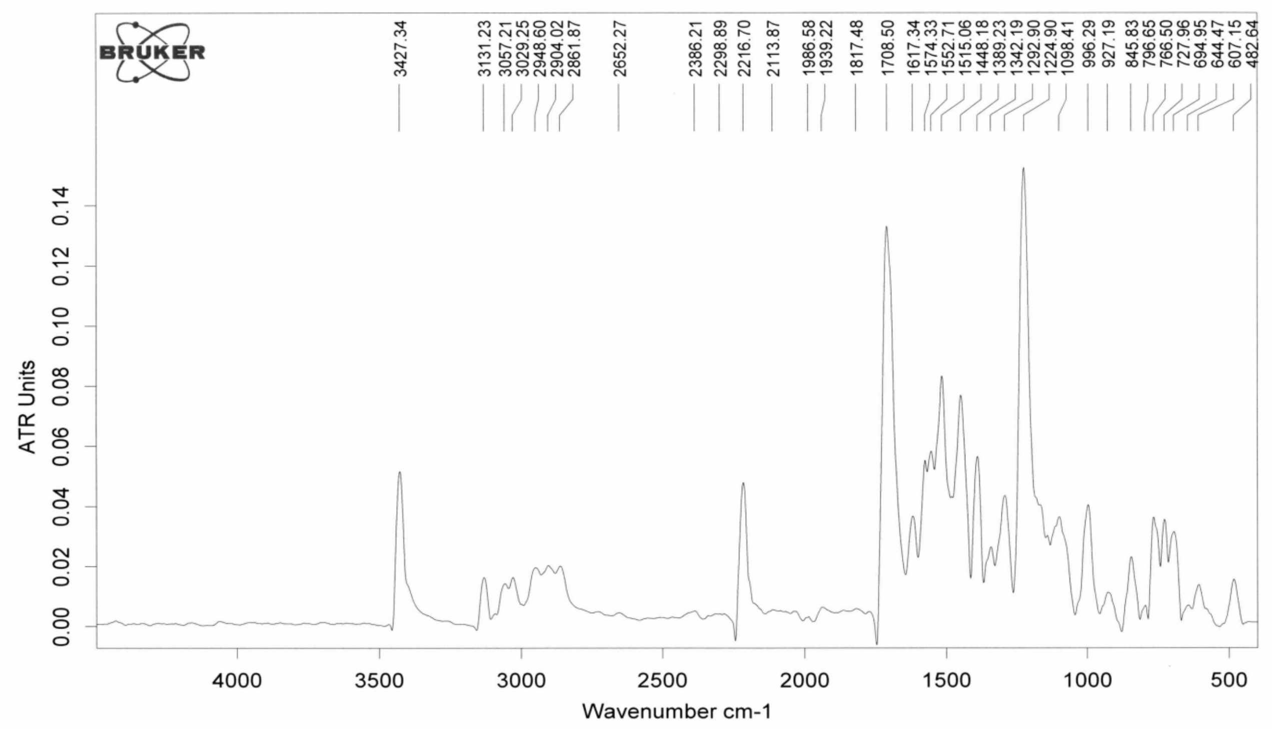

Figure S4. The IR spectrum of pyrrole **4c**

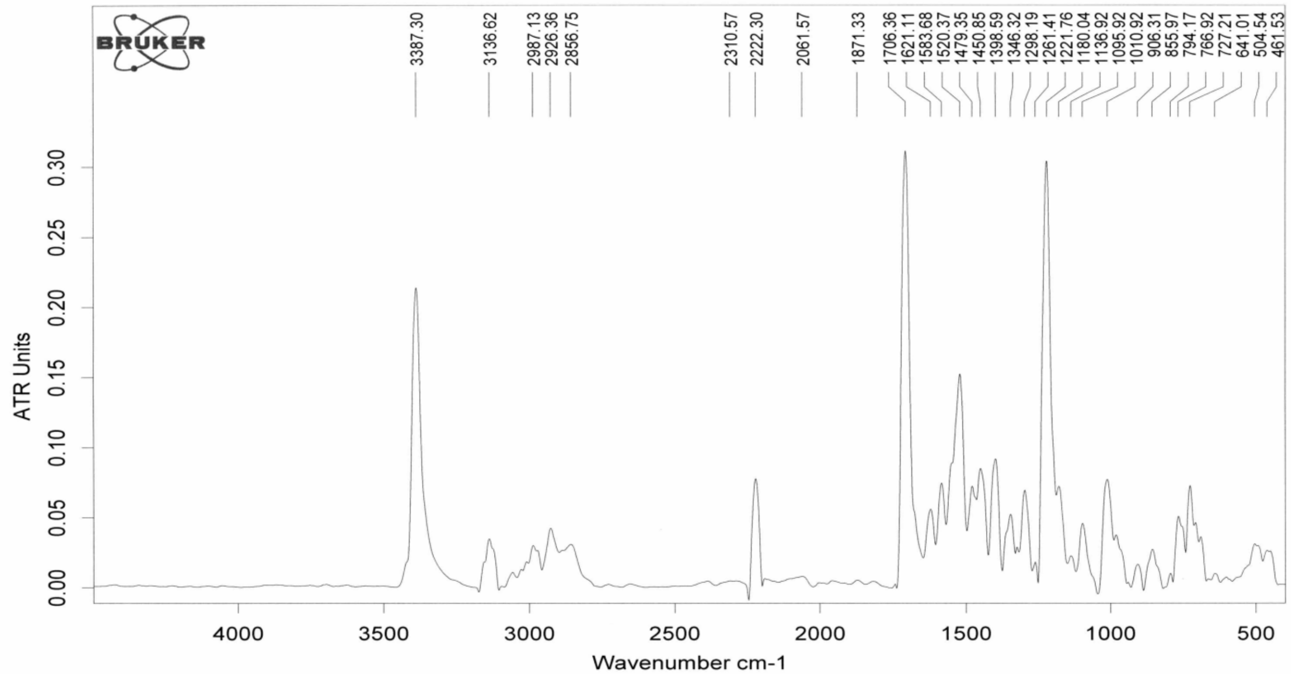

Figure S5. The IR spectrum of pyrrole **4d**

2. **The ^{1}H-NMR and ^{13}C-NMR spectra**

The NMR spectrum of 1-benzyl-5,6-dimethyl-3-cyanomethylbenzimidazolium bromide **(2c)**

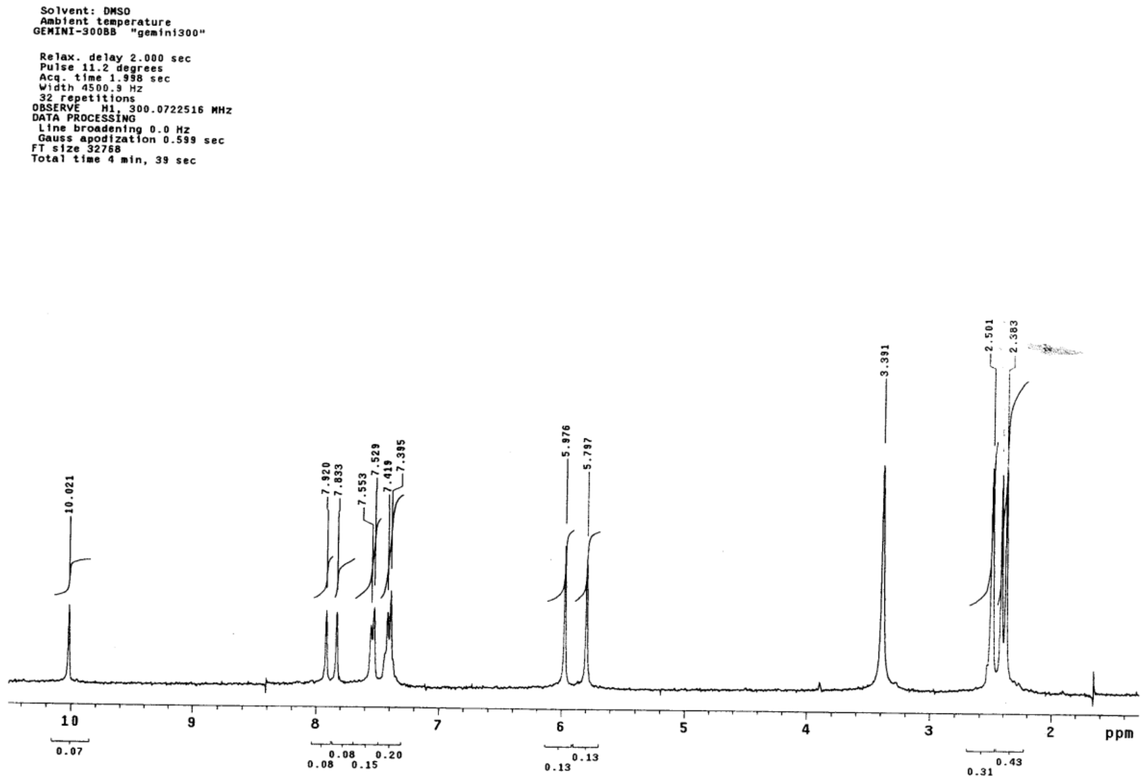

Figure S6. The ^{1}H-NMR spectrum of salt **2c**

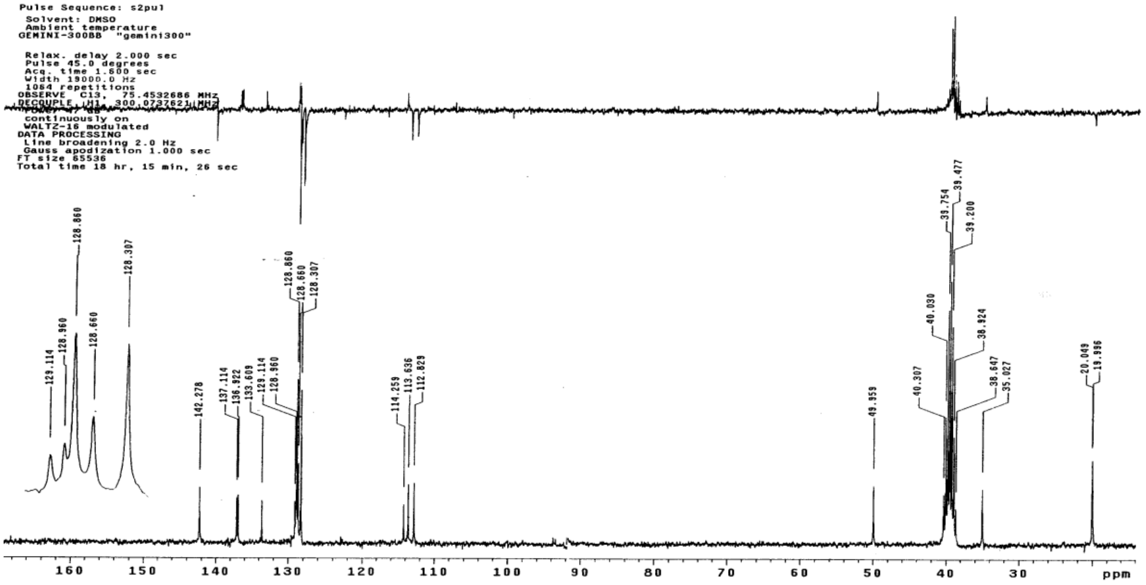

Figure S7. The ^{13}C-NMR spectrum of salt **2c**

The NMR spectrum of 4-benzoyl-2-cyano-1-(2-benzylamino-phenyl)pyrrole **(4a)**

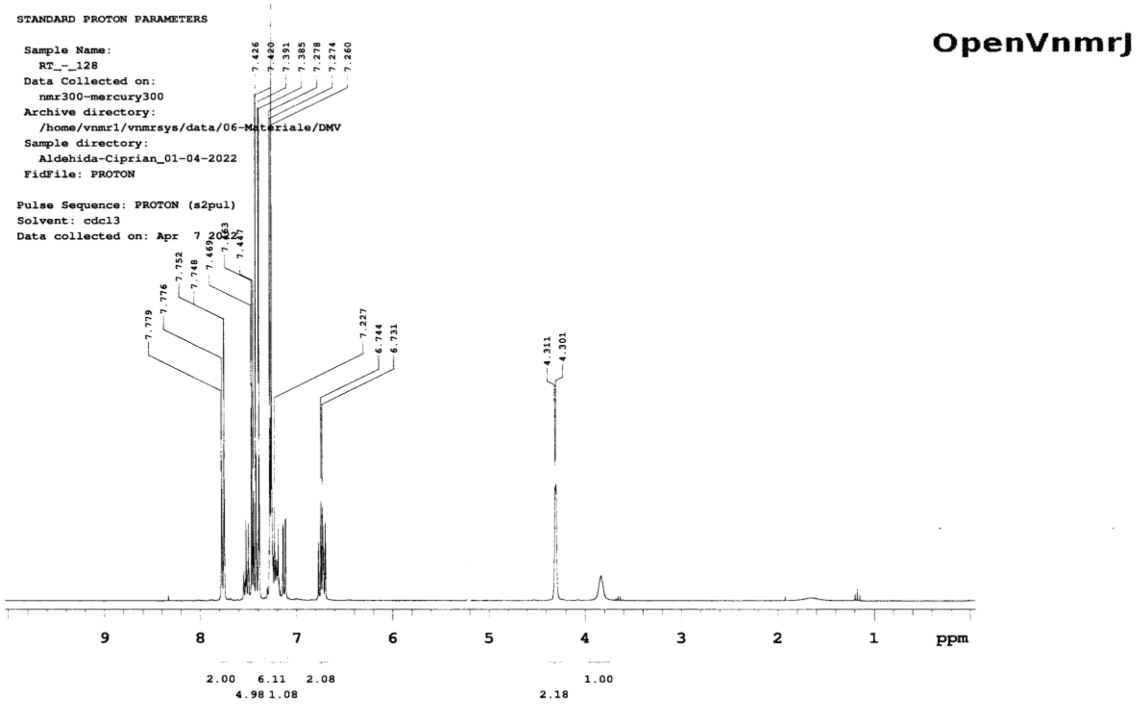

Figure S8. The ^{1}H-NMR spectrum of pyrrole **4a**

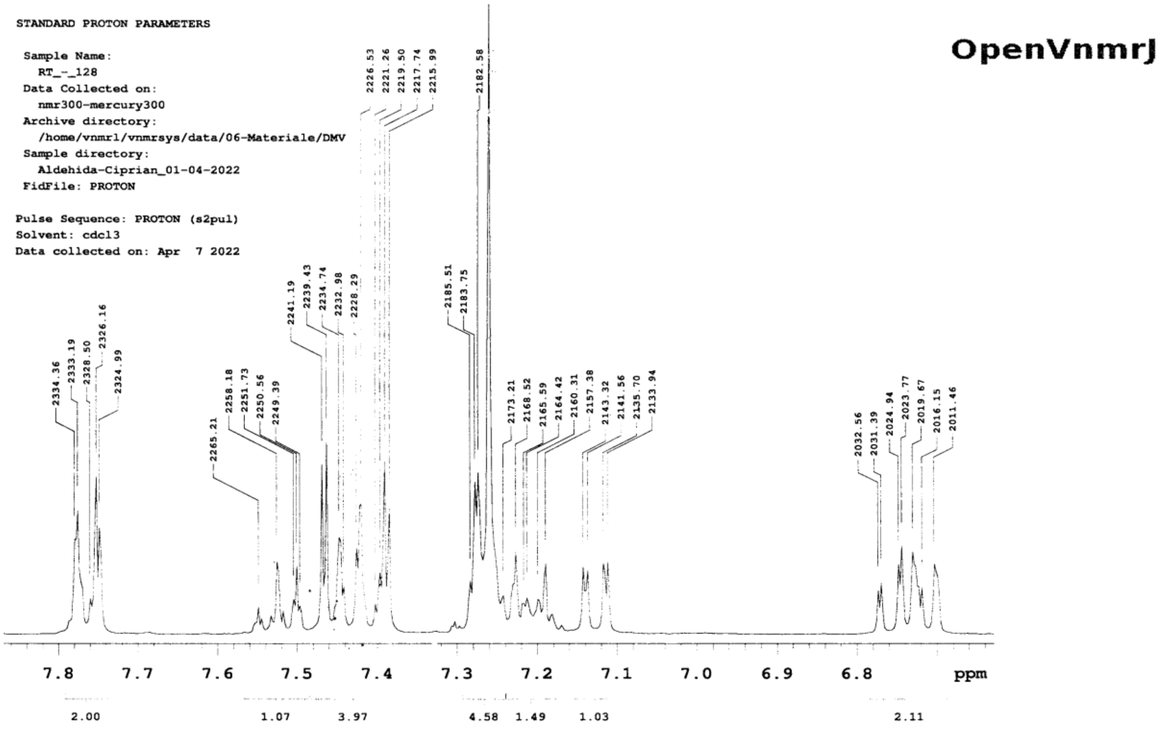

Figure S9. Detail of the ^{1}H-NMR spectrum of pyrrole **4a**

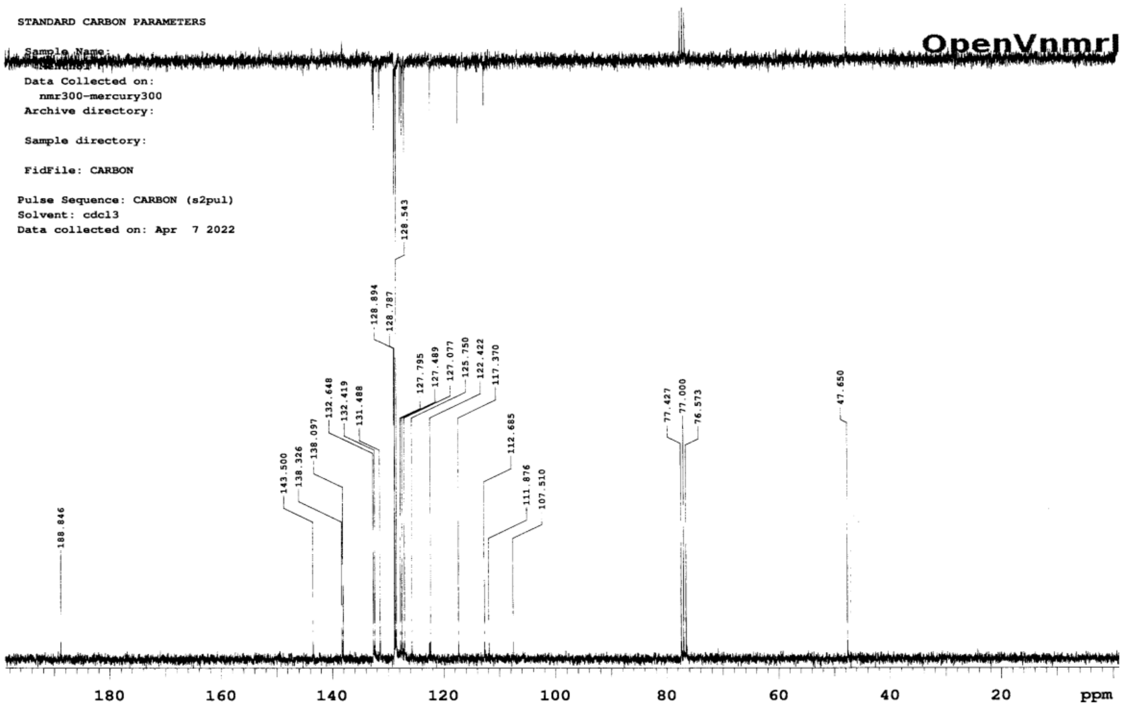

Figure S10. The ^{13}C-NMR spectrum of pyrrole **4a**

The NMR spectrum of 4-benzoyl-2-cyano-1-[2-(4-methylbenzylamino)phenyl)]pyrrole **(4b)**

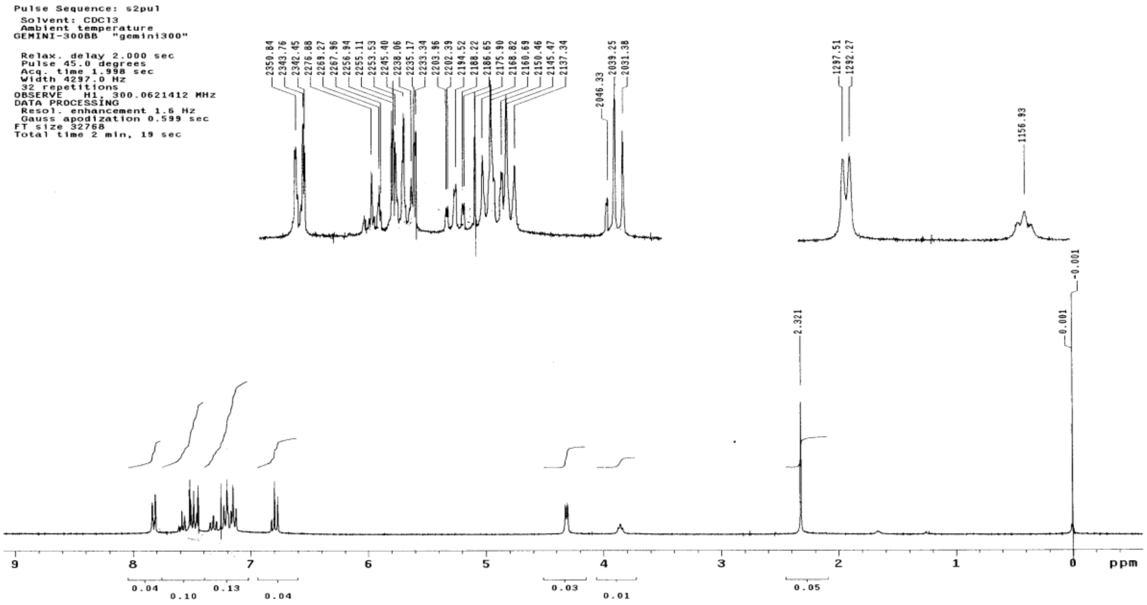

Figure S11. The ^{1}H-NMR spectrum of pyrrole **4b**

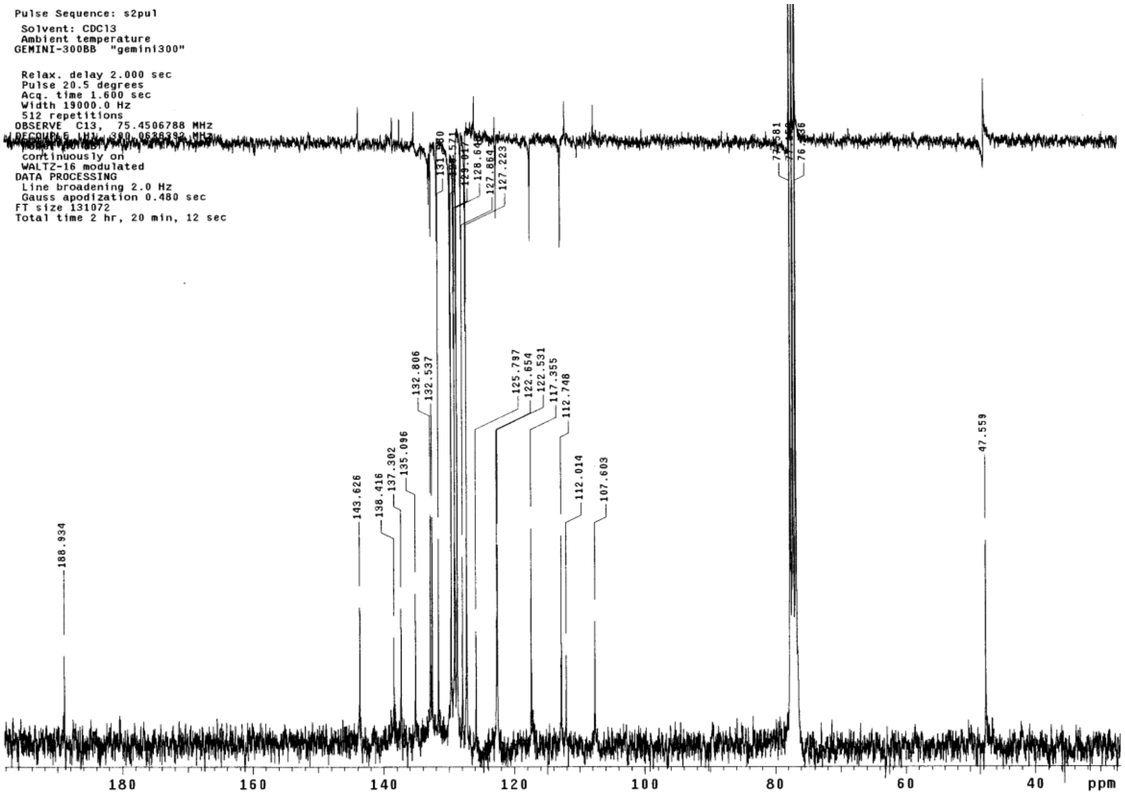

Figure S12. The ^{13}C-NMR spectrum of pyrrole **4b**

The NMR spectrum of methyl 1- [(2-benzylamino-4,5-dimethyl)phenyl]-2-cyanopyrrole-4-carboxylate **(4c)**

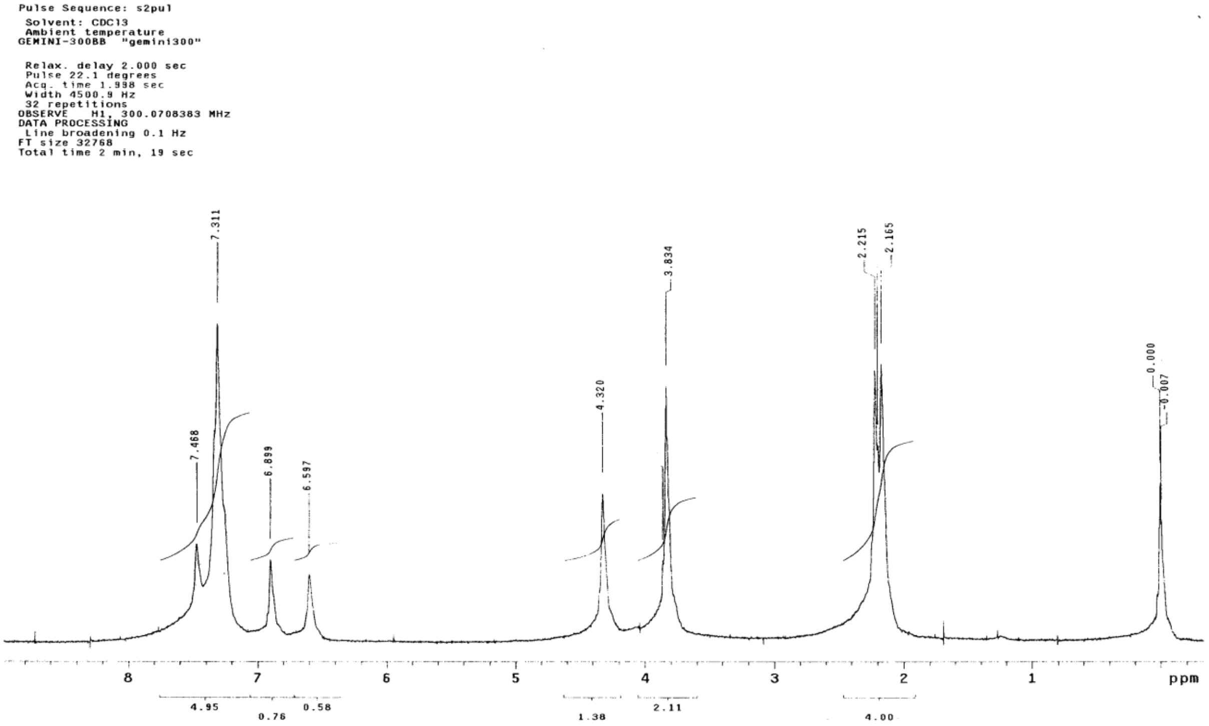

Figure S13. The ^{1}H-NMR spectrum of pyrrole **4c**

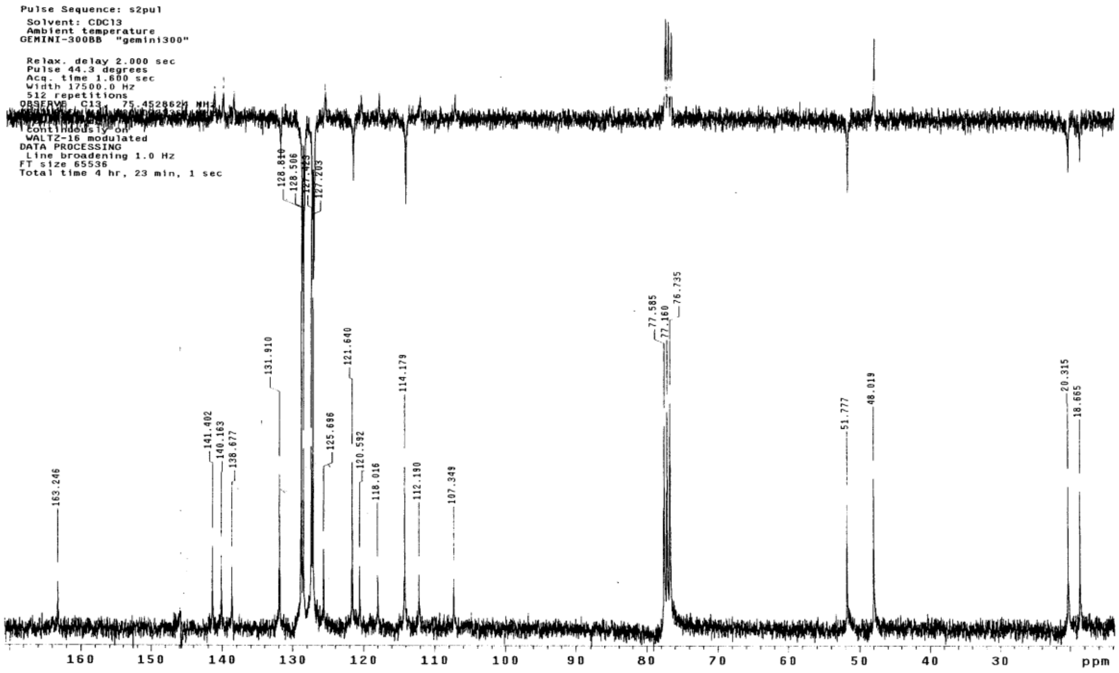

Figure S14. The ^{13}C-NMR spectrum of pyrrole **4c**

The NMR spectrum of ethyl 1- [(2-benzylamino-4,5-dimethyl)phenyl]-2-cyanopyrrole-4-carboxylate **(4d)**

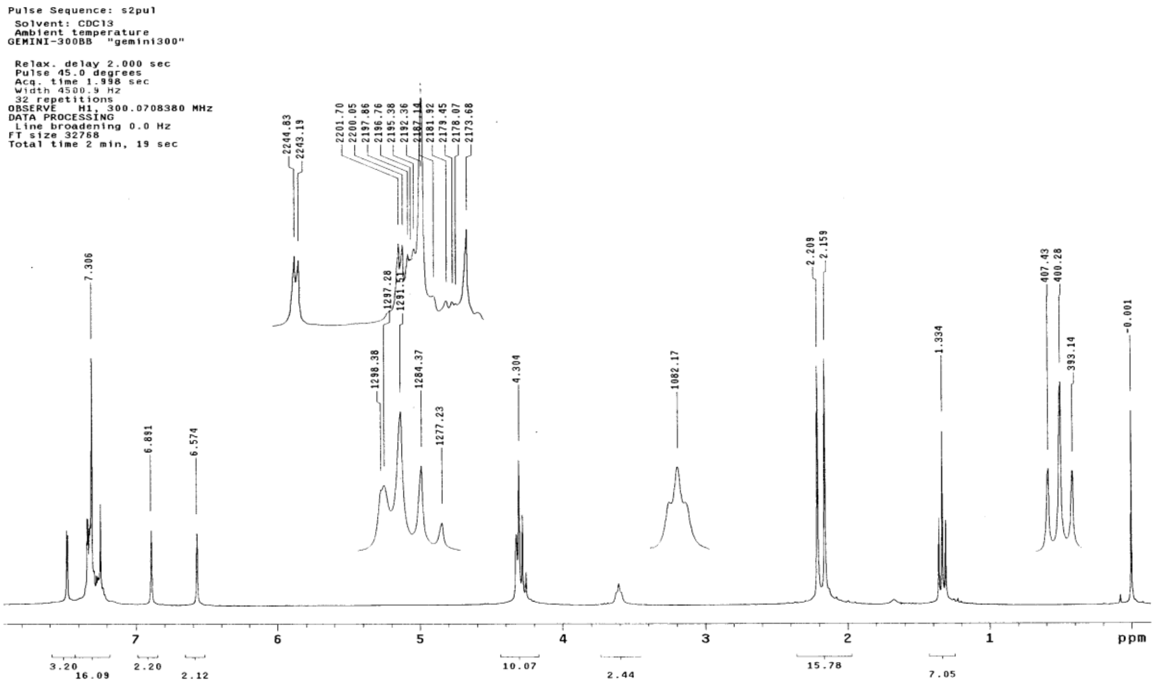

Figure S15. The ^{1}H-NMR spectrum of pyrrole **4d**

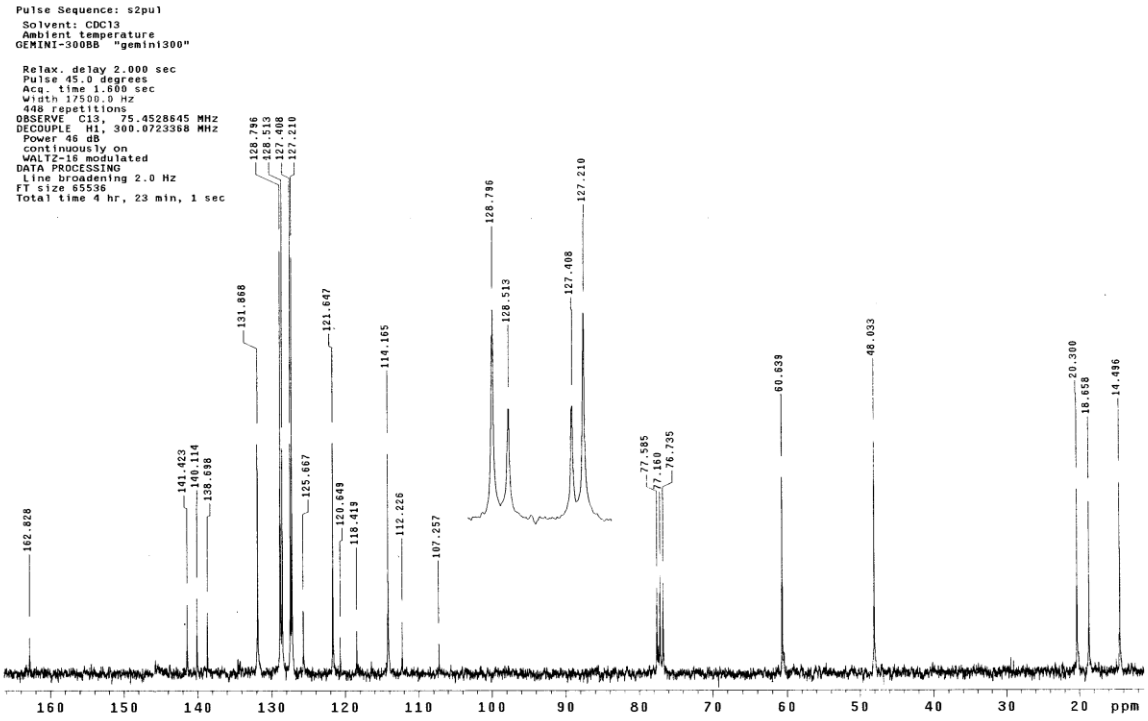

Figure S16. The ^{13}C-NMR spectrum of pyrrole **4d**
